# Metabolic Adaptation Processes That Converge to Optimal Biomass Flux Distributions

**DOI:** 10.1371/journal.pcbi.1004434

**Published:** 2015-09-04

**Authors:** Claudio Altafini, Giuseppe Facchetti

**Affiliations:** 1 Division of Automatic Control, Dept. of Electrical Engineering, Linköping University, Linköping, Sweden; 2 John Innes Centre, Norwich, United Kingdom; Hellas, GREECE

## Abstract

In simple organisms like *E.coli*, the metabolic response to an external perturbation passes through a transient phase in which the activation of a number of latent pathways can guarantee survival at the expenses of growth. Growth is gradually recovered as the organism adapts to the new condition. This adaptation can be modeled as a process of repeated metabolic adjustments obtained through the resilencings of the non-essential metabolic reactions, using growth rate as selection probability for the phenotypes obtained. The resulting metabolic adaptation process tends naturally to steer the metabolic fluxes towards high growth phenotypes. Quite remarkably, when applied to the central carbon metabolism of *E.coli*, it follows that nearly all flux distributions converge to the flux vector representing optimal growth, i.e., the solution of the biomass optimization problem turns out to be the dominant attractor of the metabolic adaptation process.

## Author Summary

In modeling metabolic networks, concepts like biomass optimization are often used to determine flux distributions of simple organisms such as *E.coli*. Although they often give good results in practice, they normally rely on heuristic considerations like “evolution has tuned metabolic fluxes to optimize growth, hence optimizing growth gives reasonable fluxes”. The main result of this paper is to show that metabolic adaptation naturally leads to optimal growth, in the sense that the flux distribution associated to optimal growth is the dominant attractor of the fitness landscape of the metabolic adaptation process.

## Introduction

Constraint-based computational methods such as Flux Balance Analysis (FBA) are nowadays widely used when investigating metabolism of bacteria and other simple unicellular organisms [1, 2]. Within the framework of FBA, a commonly accepted hypothesis is that biomass production has a special role: evolution has shaped cellular metabolism of these organisms so as to optimize growth, hence if growth is used as objective function of an optimization problem, the vector of fluxes found in correspondence of the optimum represents a plausible flux distribution for the organism. Although such a criterion is phenomenological, it is reasonable, and indeed the fluxes constructed by FBA methods describe well the empirical fluxes observed in many experimental situations, dealing with wild type organisms [3], knockout mutants [4], engineered strains, screenings of drugs [5], nutrient shifts [6] or stress responses.

For bacteria like *E.coli*, the short-term metabolic response to genetic and environmental perturbations is characterized by a growth arrest and by the activation of a number of latent pathways, a strategy which can favor the survival of the organism at the expenses of efficient biomass production [4, 6, 7]. This activation is however only transient, and most latent reactions become resilenced as the microorganism adapts to the new condition [7–9]. Although experimental data describing this adaptation process at metabolic, genomic, gene expression and proteomic level are starting to appear [6–12], it is still unclear how this dynamical recovery is implemented by the organism. From the experimental data one can deduce for instance that the FBA criterion is inadequate to describe the transient response, but that it can still be used to characterize the end-point of the metabolic adaptation [4, 6]. The two main criteria proposed in the literature to describe the metabolic response to a perturbation are MOMA (Minimization Of Metabolic Adjustment [13]) and ROOM (Regulatory On/Off Minimization [14]). Both capture the idea that metabolism tends to minimize the adjustment with respect to the pre-perturbation fluxes at the expenses of growth, and for both criteria this results in a number of non-essential reactions being activated, which is coherent with the aforementioned experimental evidence [7, 8]. However, these methods can provide only a static snapshot of the early adjustments that follow a perturbation. Attempts to model the dynamical changes happening during adaptation have been made for example using kinetic models [15] or combining pseudo steady-states of FBA with kinetic models as in dynamical FBA [16], see [17] for an overview. Other types of proposals include the incorporation of extra time-dependent constraints in the model, representing for instance molecular crowding [18] or other growth-limiting factors [19]. An alternative to adding kinetic parameters or constraints is to combine multiple datasets, such as gene expression [10, 20] and/or proteomic [12], see [21, 22] for reviews. The transcriptional or translational information obtained in this way can be used to tune the constraints of an FBA model, leading to improved matches with empirically observed fluxes [12, 20]. None of these methods is however able to provide a systematic interpretation of how and why the organism accomplishes the adaptation, let alone to propose a mathematical principle combining adaptation and FBA.

**Fig 1:**
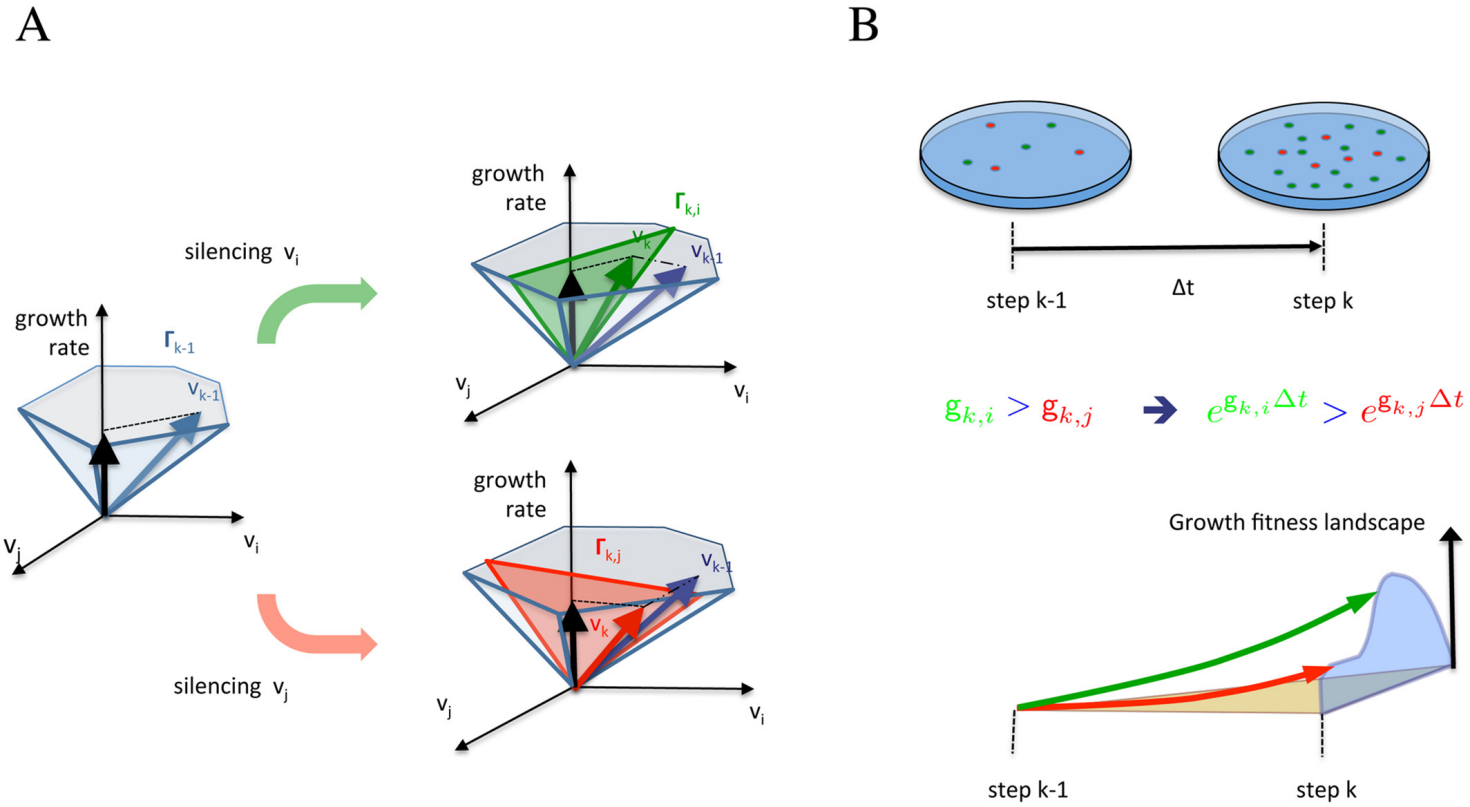
Metabolic adaptation: sketch of the process of resilencing and adjustment on the vector of fluxes. A: At step *k*, two possible resilencings are the reactions *v*_*i*_ and *v*_*j*_. Each choice gives a different reduction of the polytope Γ_*k*_ (resp. green and red) and a different MOMA projection of the current vector of fluxes **v**_*k*−1_ to the new polytope. B: Consequently also the growth rate of the two phenotypes is different and this difference is amplified in the time interval Δ*t*. Putting together all possible choices at step *k*, one gets the fitness landscape induced by the resilecings. At the end of the time interval Δ*t*, the fitness landscape gives rise to selection probabilities which have the form of a Boltzmann distribution.

A possible way to obtain a dynamical description of metabolic adaptation is proposed in [23]. Starting from a non-adapted progenitor metabolism, a population of phenotypes is obtained through the resilencing of a single reaction (i.e., letting the corresponding flux become negligible). If the growth rate of the different phenotypes is taken as measure of fitness, then a selection biased towards the fittest phenotypes favors the recovery of growth, see Fig 1. If the procedure is iterated, then a Markov chain is obtained. Since at each step of the chain the metabolism of the selected phenotype differs from its predecessor only for a single silenced reaction, it can be seen as a short-term adjustment and computed through a MOMA. For the central carbon metabolism, the resulting process of iterated metabolic adjustments leads rapidly to metabolic adaptation of the microorganism. It is shown in [24] that this model can be used to describe a series of experimental results dealing with adaptation to single carbon sources of various *E.coli* knockout strains [6]. In particular, it allows to achieve a good agreement with both the experimental growth rates and measured flux data reported in [6, 8].

The aim of this paper is to take the approach one step further, by showing that for the core metabolism of *E.coli*, the Markov chains of recursive resilencings constructed in this way exhibit a single dominant end-point flux vector, and that this vector corresponds to one of the FBA optima, namely the *parsimonious enzyme usage FBA* optimum (pFBA, minimizing the number of fluxes [12]).

To do so, we compute a large number of trajectories for our Markov chains from random initial conditions, and show that they tend to become absorbed into the pFBA flux distribution or at least to become highly correlated with it. More specifically, the chain of pseudo steady-state fluxes computed through the adjustments that follow the resilencings steers the vast majority of all admissible flux vectors towards alignment with the pFBA vector, regardless of the norm (and growth rate) achieved by the flux vectors at the end-point of the process.

In dynamical systems terms, we can say that the pFBA flux vector constitutes the dominant attractor of the fitness landscape associated to the process of metabolic adaptation. The fact that the single dominant peak of this landscape corresponds to the pFBA flux distribution sheds a novel perspective on FBA optimization, and may contribute to turning this phenomenological argument into a rigorous mathematical model.

## Methods

### FBA and pFBA

In FBA [2], the polytope of admissible steady state metabolic fluxes is represented by

Γ={v:Sv=0,l⩽v⩽u},

where **v** is the vector of fluxes, of lower and upper bounds **l** = [ℓ_1_ … ℓ_*n*_] and **u** = [*u*_1_ … *u*_*n*_], and *S* is the stoichiometric matrix. The FBA optimal flux vector is given by

$$v^{\text{FBA}}=\arg\max_{v\in \Gamma }\xi^{T}v,$$ (1)

where *g* = *ξ* ^*T*^ **v** is the growth rate, i.e., the linear combination of fluxes that constitutes the biomass reaction. When the optimum is degenerate, a secondary optimization criterion can be used to discriminate among equivalent optimal solutions. For example, overall enzyme investment is minimized by the pFBA solution **v** ^pFBA^, which corresponds to minimization of the sum of the (absolute values of the) fluxes [12].

### Adaptation as a Markov chain of repeated resilencing

Following [23], we assume that the adaptation dynamics form a stochastic process of recursive resilencings described by the Markov chain 𝓢_*k*_ = {**v**_*k*_, Γ_*k*_}_*k* = 0,1,2,…_, where **v**_0_ is a randomly chosen initial condition in Γ_0_ = Γ. The stochastic process can be summarized as follows. At step *k*, assume the population of bacteria has an homogeneous metabolism, i.e., all cells have the same *n*_*k*_ active reactions with the same fluxes **v**_*k*−1_ (this corresponds to a specific sampling of our Markov chains). From **v**_*k*−1_, it is possible to obtain *n*_*k*_ + 1 different phenotypes, corresponding to the resilencing of one of the enzymes (*n*_*k*_ possibilities) or to the current phenotype remaining unchanged for another step. The *n*_*k*_ possible silencings of a reaction yield the *n*_*k*_ reduced polytopes Γ_*k*,*i*_ = Γ_*k*−1_ ∩ {ℓ_*i*_ = *u*_*i*_ = 0}, *i* = 1, …, *n*_*k*_. The corresponding fluxes **v**_*k*,*i*_ are computed via a MOMA projection on these reduced polytopes:

$$v_{k,i}=\arg\min_{v\in \Gamma_{k,i}}\left\|v-v_{k-1}\right\|,\;i=1,\ldots ,n_{k},\;k=1,\;2,\ldots$$

where ‖ ⋅ ‖ is the Euclidean norm, see Fig 1 for a sketch.

Each choice of **v**_*k*,*i*_ leads to a possible growth rate: *g*_*k*,*i*_ = *ξ* ^*T*^ **v**_*k*,*i*_, *i* = 1, …, *n*_*k*_. Viable phenotypes have *g*_*k*,*i*_ > 0 while non-viable phenotypes (e.g. when an essential reaction is suppressed) have *g*_*k*,*i*_ = 0. In what follows these growth rates will be placed on the diagonal of a fitness matrix

$$G_{k}=[\begin{array}{cccc} g_{k,0}\\ & g_{k,1}\\ & & \ddots \\ & & & g_{k,n_{k}} \end{array}]$$

where *g*_*k*,0_ represents the current growth rate.

### Selection probabilities as solutions of a replicator equation

To the *n*_*k*_ + 1 possible choices *g*_*k*,*i*_, it is possible to associate selection probabilities through a basic replicator equation which uses the *g*_*k*,*i*_ as fitness function. Denote *p*_*k*,*i*_, *i* = 0, 1, …, *n*_*k*_, the probabilities (or frequencies) associated to the *g*_*k*,*i*_. If Δ*t* is the time duration of each step, then the replicator equation is

$${\dot{p}}_{k}=G_{k}p_{k}-\phi \left(p_{k}\right)p_{k}\;\tau \in \left[0,\;\Delta t\right],$$ (2)

where

$$p_{k}=[\begin{array}{c} p_{k,0}\\ p_{k,1}\\ \vdots \\ p_{k,n_{k}} \end{array}]$$

and $\phi ({\text{p}}_{k})=\sum_{i=0}^{n_{k}}g_{k,i}p_{k,i}$ is the average fitness. The explicit solution of Eq (2) can be expressed as a Boltzmann distribution, see S1 Text for the details. In synthesis, in the two cases we can distinguish (sketched in Fig. B of S1 Text) one gets for the selection probabilities:

1. *uniform priors*: at the begin of the time interval the selection probability is **p**_*k*_(0) = **1**/(*n*_*k*_ + 1), where $\text{1}={\left[1\ldots 1\right]}^{T}$, i.e., all choices are equiprobable. In this case the Boltzmann distribution for the selection probabilities at the end of the time interval is
  $$p_{k}\left(\Delta t\right)=\frac{1}{Z_{k}\left(\Delta t\right)}e^{G_{k}\Delta t}1$$
  where $Z_{k}(\Delta t)\;=\;\sum_{i=0}^{n_{k}}e^{g_{k,i}\Delta t}$ is a partition function.
2. *non-uniform priors*: at the begin of the time interval the selection frequencies are not uniform but are themselves expressible as a Boltzmann distribution
  $$p_{k}\left(0\right)=\frac{1}{Z_{k}\left(\beta_{k}\right)}e^{G_{k}\beta_{k}}1$$
  where *β*_*k*_ has the interpretation of an inverse temperature. In this case, at the end of the time interval we obtain
  $$p_{k}\left(\Delta t\right)=\frac{1}{Z_{k}\left(\beta_{k}+\Delta t\right)}e^{G_{k}\left(\beta_{k}+\Delta t\right)}1.$$

A through derivation of these selection probabilities is available in the S1 Text.

### Metabolic adaptation as a completely reducible Markov chain

**Fig 2:**
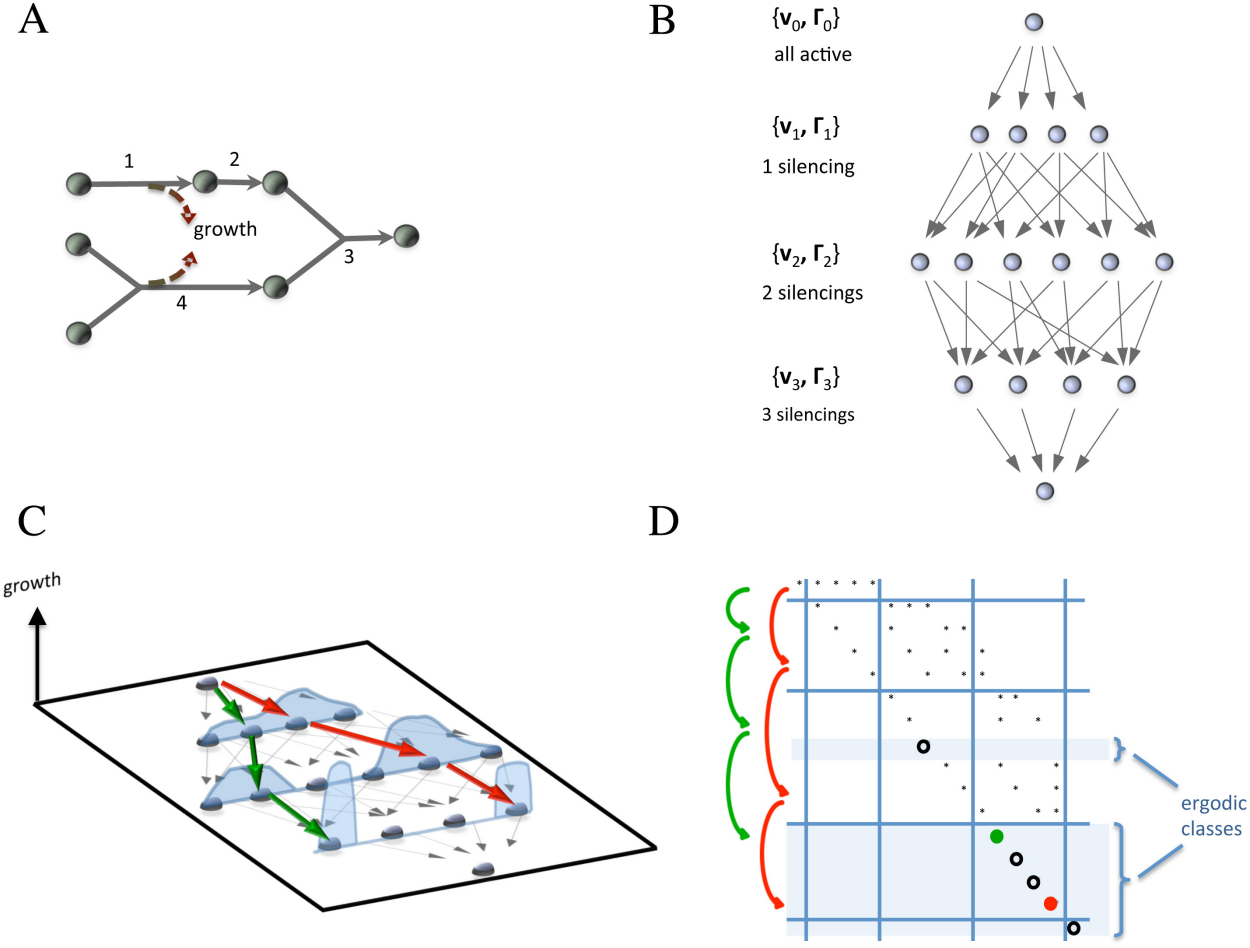
Sketch of a resilencing Markov chain for a toy metabolic network of *r* = 4 reactions. For the metabolic network in A, the 2^4^ states of the projected Markov chain (i.e., the 16 possible on/off combinations of the 4 reactions) are shown in B. C: The fitness landscape in terms of growth rate (which depends also on the flux vector **v**) and two trajectories represented through their state transitions. D: The transition matrix of the projected Markov chain with its triangular structure and the ergodic classes, corresponding to the rows having a 1 on the diagonal and 0 elsewhere. The 0-growth ergodic classes have probability ∼ 0 of absorbing trajectories.

In both cases described above **p**_*k*_(Δ*t*) has the meaning of transition probability between the current state 𝓢_*k*−1_ = {**v**_*k*−1_, Γ_*k*−1_} and the possible states achievable at the *k*-th step 𝓢_*k*,*i*_ = {**v**_*k*,*i*_, Γ_*k*,*i*_}, i.e., $p_{k,i}=\mathbb{P}(X_{k}={𝒮}_{k,i}\;|\;X_{k-1}={𝒮}_{k-1})$, *i* = 0, 1, …, *n*_*k*_. Since the fluxes **v**_*k*,*i*_ can take any value between lower and upper bound, the corresponding transition matrix is infinite dimensional. However, in order to understand the properties of the stochastic process we are considering, it is useful to look at its projection over the subspace of active reactions (i.e., over the binary equivalent of the polytope Γ_*k*_). In terms of this projection, the possible states of the Markov chain are the 2^*r*^ possible combinations of the *r* reactions of the network, see Fig 2 for a toy example with *r* = 4. Denote 𝓩_1_, …, 𝓩_2^*r*^_ these discrete states and *P*_*ij*_ = ℙ(*X*_*k*_ = 𝓩_*i*_ ∣ *X*_*k*−1_ = 𝓩_*j*_) the corresponding transition probabilities. As in our model the resilencings are irreversible, *P* is triangular, i.e., it is completely reducible, see Fig 2D. Since in reality *P* is the result of a projection, it is *P* = *P*(**v**), i.e., the exact transition probabilities **p**_*k*_ depend on the values of the fluxes and hence on **v**_0_. However, even in the complete model the fully reducible structure is preserved. In particular it follows that a certain number of states 𝓩_*i*_ must correspond to ergodic classes, i.e., “absorbing” states in which the Markov chain stabilizes. Full reducibility implies that each ergodic class is composed of a single state. Periodic chains of states are impossible.

## Results

The metabolic adaptation process described in Methods and in Fig 1 is applied to the network that describes the central carbon metabolism of *E.coli* [25]. In order to do this, a large number of realizations of the Markov chain is produced. Even in a network of modest dimensions like that of *E.coli* central metabolism (*r* = 95 reactions, see S1 Text for details), the number of possible discrete states 𝓩 of the Markov chain is enormous (2^95^ ∼ 10^28^), hence numerical computations are necessarily limited to a fraction of all possible trajectories. For this study, some 10^5^ trajectories have been generated, starting from randomly chosen initial conditions in the polytope of admissible fluxes Γ and using various forms for the priors.

**Fig 3:**
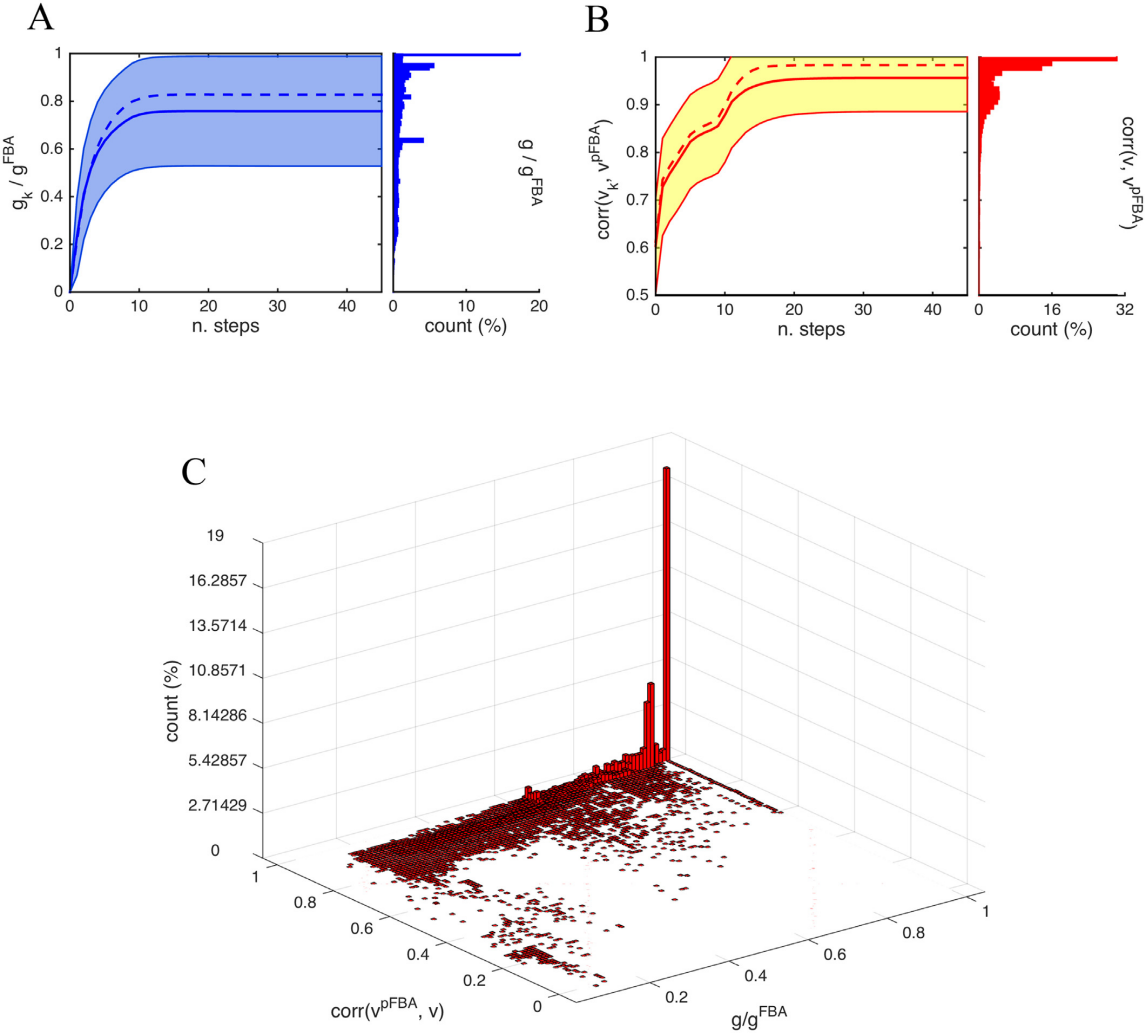
Metabolic adaptation: growth rate and correlation. A: Mean ± std of the growth rate during adaptation computed over 10^5^ sample trajectories (solid lines, the dash line represents the median). The end-point of the trajectories is shown in the vertical histogram. The mean value at absorption is $\frac{\left\langle g\right\rangle }{g^{\text{FBA}}}=0.76$, and the median 0.83. The histogram is significantly skewed (z-test, p-value 0.05): around 47% of the trajectories reach a growth rate of 0.85*g* ^pFBA^. B: Mean ± std of the correlation between **v**_*k*_ and **v** ^pFBA^ during adaptation (solid lines). At absorption, the mean of the correlation is 0.96. As can be seen in the histogram of the end-points, the distribution is highly skewed towards maximal correlation, with 68% of end-points above the mean. In fact the median is 0.98 (dashed line). C: The 3D histogram shows the correlation between **v** ^pFBA^ and the end-point of the trajectories **v** versus the growth rate *g* reached by **v**. Of the trajectories reaching *g*/*g* ^FBA^ > 0.85, 99% have correlation ≥ 0.85.

Given the irreversibility of the resilencing, the number of steps required to reach an end-point state can be computed from the trajectories. A trajectory is considered absorbed into an end-point state (i.e., it has reached a local maximum of the fitness landscape) when no further silencing happens for 5 consecutive steps. With this stopping condition, the expected time until absorption of our trajectories is 24.6 ± 4.1 steps. Except for pathological initial conditions (those missing some essential reactions), all trajectories stabilize to flux vectors of positive growth. Nearly 20% of all trajectories reach the maximal growth computed by the FBA criterion, *g* ^FBA^, and for nearly 50% of all trajectories the growth at the end-point is ≥ 0.85 *g* ^FBA^, see Fig 3. The remaining 50% of trajectories are more or less uniformly distributed in the interval 0.2 < *g*/*g* ^FBA^ < 0.85. Much more remarkable is the correlation between the end-point flux distribution **v** and the flux distribution given by the pFBA criterion **v** ^pFBA^: the mean of the correlation is 0.96 and the median is 0.98, with 88% of all trajectories achieving a correlation of at least 0.9, see Fig 3. The meaning of this result is that nearly all initial conditions in Γ tend to become aligned with the flux distribution **v** ^pFBA^, regardless of the biomass they can produce, see Fig. A of S1 Text for a sketch.

The time evolution of the 3D histogram of Fig 3 during adaptation is shown in Fig. C of S1 Text. It can be seen that while randomly chosen initial conditions in Γ usually give a zero-growth, already with the first silencings growth starts to recover, and gradually improves in the first 10 steps of the Markov chain. During the transient, no significant intermediate peak is visible, meaning that many different routes are explored by the trajectories. After ∼ 10 steps, the high correlation / high growth peak starts to appear, and rapidly becomes dominant.

Examples of the resulting trajectories are shown in Figs. D-F of S1 Text. For instance, the first row of Fig. D of S1 Text shows a set of trajectories originating from the same random initial condition, all converging towards **v** ^pFBA^, although through slightly different paths. None of the trajectories of the second row of Fig. D of S1 Text instead achieves a growth rate higher than 0.75*g* ^FBA^. However, all of the end-points flux vectors become aligned with **v** ^pFBA^ (correlation higher than 0.97). In this case the two values of *g* reached by the trajectories correspond to two different ergodic states, as can be seen by the grouping of the number of active reactions eventually reached. It should be observed how for this phenotype of non-optimal growth the number of reactions is much less than for **v** ^pFBA^. This is indeed a constant pattern in our metabolic adaptation strategy. As can be seen in Fig. G of S1 Text, at the end-point the number *R* of active reactions of **v** and *g* are positively correlated: for strains that have sub-optimal growth more resilencings are possible i.e., more directions with slow but positive Δ*g* exist and are explored. In fact, Fig. G of S1 Text shows that indeed the length *L* of a trajectory is inversely correlated with the growth *g* of **v**.

Other than the peak at high correlation / high growth, Fig 3 does not show any other sufficiently significant peak (and nor does Fig. C of S1 Text). It is however worthwhile observing that a small fraction of trajectories is steered towards flux distributions of maximal growth different from **v** ^pFBA^, i.e., to alternative FBA optima. An example of such trajectory is shown in Fig. E of S1 Text (bottom row): while most of the trajectories converge to **v** ^pFBA^, a few do not (one is shown in green), and stabilize in an alternative FBA flux vector of correlation 0.75 with **v** ^pFBA^. Cases like this lead to a correlation corr(**v** ^pFBA^, **v**_*k*_) which decreases when **v**_*k*_ falls into the basin of attraction of a local maximum other than **v** ^pFBA^. In the ensemble of the trajectories, however, these situations are unfrequent: if we look at the average of all trajectories, corr(**v** ^pFBA^, **v**_*k*_) is always monotonically increasing, regardless of the final *g* achieved, see Fig 3 and Fig. H of S1 Text. Similarly, also *g* is monotonically growing on the vast majority of the trajectories (Fig. I of S1 Text).

Interestingly, if we start relaxing the assumption of irreversibility that characterizes a large fraction of the metabolic reactions (49 of the 95 reaction are irreversible in our network), then the convergence rate to **v** ^pFBA^ quickly decreases, in favor of other **v** ^FBA^, see Figs. J-L of S1 Text. In particular, when all reactions are considered as reversible, then the correlation between **v** ^pFBA^ and **v** at absorption is completely lost, although optimal biomass is still achieved by most trajectories, see Fig. L of S1 Text.

**Fig 4:**
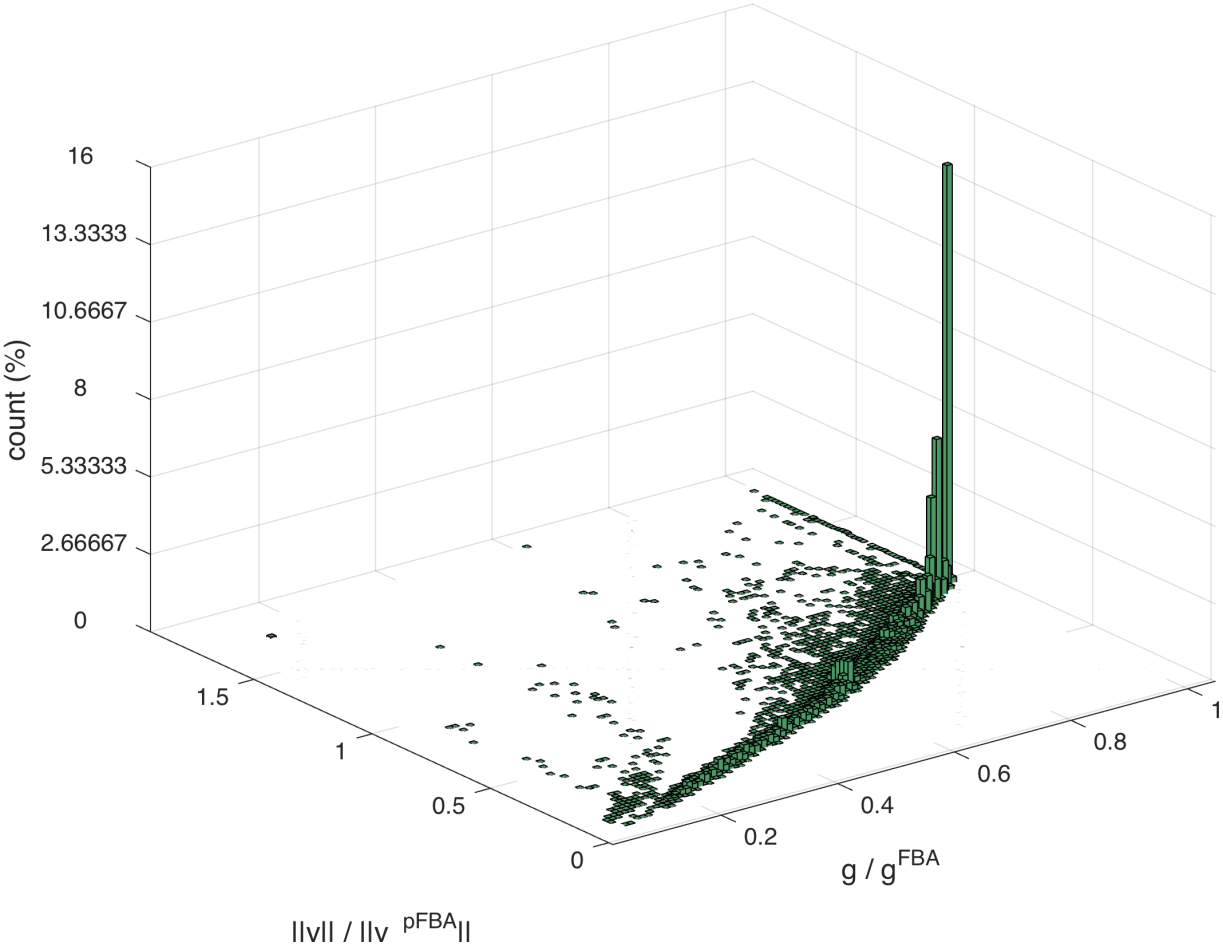
Norm of v vs. growth rate. The histogram shows that the growth rate achieved by the adaptation process is proportional to the ratio $\frac{||\text{v}||}{||{\text{v}}^{\text{pFBA}}||}$ at the time of absorption. “Short” **v** cannot give maximal growth.

It follows directly from the linearity of the expression for the biomass that when a trajectory **v** becomes aligned with **v** ^pFBA^, the growth rate it can produce depends on the norm of **v**. In fact, Fig 4 shows that there is a sharp direct proportionality between ‖**v**‖ at ergodicity and *g*: when the recursive process of silencings and adjustments leads to a **v** which is smaller in norm than **v** ^pFBA^, also the corresponding *g* will be smaller than *g* ^FBA^.

In order to understand when an initial condition can lead to an end-point **v** of norm comparable to **v** ^pFBA^, one can look at how many of the bounds that delimit the polytope of admissible fluxes at step *k*, Γ_*k*_, become active during the adaptation (i.e., a flux for a reaction becomes equal to one of its lower or upper bounds). Fig. N of S1 Text shows that in the early part of the adaptation, trajectories that do not achieve high growth (which, from Fig 4, correspond to trajectories having ‖**v**‖ < ‖**v** ^pFBA^‖) tend to saturate less than those achieving higher growth. Hence fluxes that tend to stay in the interior of the polytope rarely will reach a high ‖**v**‖. From Fig. O of S1 Text it can be observed that the difference in active bounds concerns mostly certain specific pathways: in strains achieving high growth, uptake bounds on many exchange reactions tend to become saturated in the early transient, and so do upper bounds of pyruvate metabolism, signs of a more efficient use of the available resources. Coherently, Fig. P of S1 text says that high growth is achieved when TCA cycle and pentose phosphate pathway remain fully functional during adaptation. Notice that uptake bounds of gluconeogenic carbon sources such as acetate are almost never saturated in the high growth trajectories.

## Discussion

Mathematically, the dynamical model used in this paper to describe metabolic adaptation has many aspects in common with standard evolutionary models based on natural selection [26]. The only extra assumption we require is that the “selection” that leads to adaptation occurs only through the silencing of non-essential reactions. That a multitude of latent pathways becomes active after an environmental perturbation is a known fact experimentally [7, 8]. That during adaptation these low-yield pathways tend to become resilenced is also a commonly accepted hypothesis [8, 27], supported for example by gene expression data. It has in fact been observed that e.g. after a change of carbon source a major rearrangement occurs at gene expression level, with more than 10^3^ genes differentially expressed [8]. A similar pattern is observed also in response to a wide variety of stress factors [7]. After a strain has adapted to the new condition, however, most differentially expressed genes have returned to their baseline level, and so is probably the concentration of the corresponding enzymes.

As shown in [7], different stress responses elicit early metabolic responses that are less stereotypical than those observed at gene expression level. When growth is recovered, however, the metabolic profiles in the various cases show a high similarity. This picture is coherent with the presence of an attractor in flux space, which can compensate for possibly widely different flux distributions right after a perturbation. For metabolic responses such as the stress responses of [7], it is unclear how to include the direct effect of the perturbation on the metabolic fluxes of an FBA model. To cope with this fact, in our Markov chains the initial condition for the flux vector, **v**_0_, is chosen randomly in the polytope Γ, which implies that at the begin of the Markov chain most reactions are already active.

When instead the effect of a specific perturbation can be explicitly included in the FBA model, then the Markov chains can be used to investigate also the early stages of the transient, with the activation of the latent pathways. This is the point of view taken in [24], where the experimental setting of [8] is considered. It is shown in [24] that the shift from rich medium to single carbon sources for various *E.coli* mutants can be reproduced closely by the metabolic adaptation process described in the Methods section. Proceeding in this way corresponds to fixing specific initial conditions on the Markov chains, and following the specific family of trajectories that results from them (activatory phase included). It becomes then interesting to see what happens when these “nominal” trajectories are compared to more general trajectories in which the initial fluxes **v**_0_ are randomly chosen in Γ. For glucose as single carbon source, the two types of trajectories are compared in Fig. M of S1 Text. As can be seen, for all 4 mutant strains there is a high correlation between the end-points achieved by the flux vectors, meaning that the specific pattern of transient activations of the latent pathways is not crucial to the achievement of the adapted flux distribution, as both types of trajectories converge towards **v** ^pFBA^. Notice how the *pgi* mutant has a secondary peak at low correlation: this corresponds to a less frequent second phenotype of lower growth, described in [8]. Such a phenotype is sometimes achieved by both the nominal trajectories of [24] and the randomly initialized trajectories computed in this paper.

A number of possible optimality criteria alternative to biomass optimization have been investigated in the literature [2, 28–30]. Common choices are for example maximization of yield (instead of biomass), maximization of ATP, minimization of overall intracellular flux (i.e., minimum enzyme investment), minimization of redox potential, etc. In [29] a thorough analysis of their coexistence/complementarity is carried out. By using reaction resilencing to progressively adjust the metabolism to the new environment, two of the most accepted among these criteria, biomass optimization and minimization of overall fluxes, are naturally combined.

It is shown in [31] that in FBA irreversibility of a large fraction of metabolic reactions is a key factor in achieving optimal flux distributions that are sparse, as our pFBA is. Indeed also for our metabolic adaptation process irreversibility is key to convergence to **v** ^pFBA^, as Figs. J-L of S1 Text clearly show. It is worth observing that when we start relaxing the assumption of irreversibility, what is lost is not the achievement of optimal growth, but only convergence to the sparsest degenerate solution of Eq (1) (i.e. **v** ^pFBA^). On the contrary, in the case of all reversible reactions the ratio *g*/*g* ^FBA^ achieved by the trajectories is even better than in Fig 3, with a mean value for *g* of 0.91*g* ^FBA^ and a median value of 0.997*g* ^FBA^, see Fig. L of S1 Text. Given that the irreversibility of the constraints follows from thermodynamic considerations [32] which are usually considered sufficiently reliable, our results provide novel evidence in favor of sparse optimal biomass solutions such as pFBA, and a novel point of view on the coexistence of optimality criteria such as biomass production and enzyme parsimony of the solution. They also confirm that the repeated resilencing process described in this paper is indeed an effective strategy for describing the recovery of growth that occurs in metabolic adaptation.

It is worth remarking that the method used in this paper is fundamentally different from a dynamical FBA [16]. In the latter, in fact, growth is used as the objective function of an optimization problem, and the adjustments of the metabolic fluxes follow the gradient direction indicated by the solution of such a problem. In our case, instead, the growth rate is only used to shape the fitness landscape of a population of possible phenotypes (corresponding to the possible silencings that can occur), but the metabolic adjustments are always computed through MOMA projections. In general, there is no *a priori* guarantee that a greedy fitness landscape constructed in this way i) may be regular; ii) may achieve maximal growth, and iii) may lead to flux distributions that resemble those of the pFBA. In our trajectories, in fact, what we observe is that the fitness landscape is rugged, but the plethora of local maxima have all a very small basin of attraction, as opposed to the global maximum which attracts around 50% of all trajectories when we count based on growth. If instead we look at normalized flux distributions then the basin of attraction of $\frac{{\text{v}}^{\text{pFBA}}}{||{\text{v}}^{\text{pFBA}}||}$ grows to 90% of all $\frac{\text{v}}{||\text{v}||}$. This tells us that for what concerns central metabolism, a procedure like the one described in this paper is substantially a monotonic process of alignement of **v**_*k*_ on **v** ^pFBA^. The robustness of the convergence is also reflected in the low sensitivity to the randomness of the Markov chains, see S1 Text and Fig. T of S1 Text for more details.

In conclusion, one can say that simple flux reorganization rules based on local fitness are sufficient to drive the cell toward a more efficient use of the metabolic resources. It is quite remarkable that most of the trajectories end up in the pFBA optimum, without knowing it, and without ever using growth rate to update metabolic fluxes (growth rate is used only for the selection probabilities in the resilencings; metabolic fluxes are always updated via MOMA). Clearly this fact provides a further evidence in favor of the FBA criterion, and one could even speculate that it provides a more fundamental principle, from which FBA follows as a corollary.

## Supporting Information

S1 text Methods. Details of the population dynamics model and of the selection probabilities. Further considerations of the method and on its applicability.
(PDF)

## References

[1] Bordbar A, Monk JM, King ZA, Palsson BO. Constraint-based models predict metabolic and associated cellular functions. Nature reviews Genetics. 2014 2;15(2):107–120. 24430943 10.1038/nrg3643

[2] Palsson BO. Systems Biology: Properties of Reconstructed Networks. Cambridge University Press; 2006.

[3] Edwards JS, Ibarra RU, Palsson BO. In silico predictions of Escherichia coli metabolic capabilities are consistent with experimental data. Nature biotechnology. 2001 2;19(2):125–130. 10.1038/84379 11175725

[4] Ibarra RU, Edwards JS, Palsson BO. Escherichia coli K-12 undergoes adaptive evolution to achieve in silico predicted optimal growth. Nature. 2002 11;420(6912):186–189. 10.1038/nature01149 12432395

[5] Folger O, Jerby L, Frezza C, Gottlieb E, Ruppin E, Shlomi T. Predicting selective drug targets in cancer through metabolic networks. Mol Syst Biol. 2011;7:501–511. 10.1038/msb.2011.35 21694718 PMC3159974

[6] Fong SS, Palsson BØ. Metabolic gene–deletion strains of Escherichia coli evolve to computationally predicted growth phenotypes. Nature genetics. 2004;36(10):1056–1058. 10.1038/ng1432 15448692

[7] Jozefczuk S, Klie S, Catchpole G, Szymanski J, Cuadros-Inostroza A, Steinhauser D, et al Metabolomic and transcriptomic stress response of Escherichia coli. Molecular Systems Biology. 2010 5;6:364 10.1038/msb.2010.18 20461071 PMC2890322

[8] Fong SS, Nanchen A, Palsson BØ, Sauer U. Latent pathway activation and increased pathway capacity enable Escherichia coli adaptation to loss of key metabolic enzymes. Journal of Biological Chemistry. 2006;281(12):8024–8033. 10.1074/jbc.M510016200 16319065

[9] Buescher JM et al Global network reorganization during dynamic adaptations of Bacillus subtilis metabolism. Science. 2012 3;335(6072):1099–1103. 10.1126/science.1206871 22383848

[10] Fong SS, Joyce AR, Palsson BØ. Parallel adaptive evolution cultures of Escherichia coli lead to convergent growth phenotypes with different gene expression states. Genome research. 2005 10;15(10):1365–1372. 10.1101/gr.3832305 16204189 PMC1240078

[11] Charusanti P, Conrad TM, Knight EM, Venkataraman K, L FN, Xie Y B amd Gao, et al Genetic basis of growth adaptation of Escherichia coli after deletion of pgi, a major metabolic gene. PLoS Genetics. 2010;6(11):e1001186 10.1371/journal.pgen.1001186 21079674 PMC2973815

[12] Lewis NE, Hixson KH, Conrad TM, Lerman JA, Charusanti P, Polpitiva AD, et al Omic data from evolved E.coli are consistent with computed optimal growth from genome-scale models. Mol Sys Biol. 2010;6. 10.1038/msb.2010.47 PMC2925526 20664636

[13] Segré D, Vitkup D, Church GM. Analysis of optimality in natural and perturbed metabolic networks. Proc Natl Acad Sci USA. 2002;99 (23):15112–15117. 10.1073/pnas.232349399 12415116 PMC137552

[14] Shlomi T, Berkman O, Ruppin E. Regulatory on/off minimization of metabolic flux changes after genetic perturbations. Proc Natl Acad Sci USA. 2005;102 (21):7695–7700. 10.1073/pnas.0406346102 15897462 PMC1140402

[15] Kotte O, Zaugg JB, Heinemann M. Bacterial adaptation through distributed sensing of metabolic fluxes. Molecular systems biology. 2010 3;6:355 10.1038/msb.2010.10 20212527 PMC2858440

[16] Mahadevan R, Edwards JS, Doyle FJ III. Dynamic flux balance analysis of diauxic growth in Escherichia coli. Biophysical journal. 2002;83(3):1331–1340. 10.1016/S0006-3495(02)73903-9 12202358 PMC1302231

[17] Antoniewicz MR. Dynamic metabolic flux analysis–tools for probing transient states of metabolic networks. Current opinion in biotechnology. 2013 12;24(6):973–978. 10.1016/j.copbio.2013.03.018 23611566

[18] Beg QK, Vazquez A, Ernst J, de Menezes MA, Bar-Joseph Z, Barabási AL, et al Intracellular crowding defines the mode and sequence of substrate uptake by Escherichia coli and constrains its metabolic activity. Proceedings of the National Academy of Sciences. 2007;104(31):12663–12668. 10.1073/pnas.0609845104 PMC1937523 17652176

[19] O’Brien EJ, Lerman JA, Chang RL, Hyduke DR, Palsson BØ. Genome-scale models of metabolism and gene expression extend and refine growth phenotype prediction. Molecular systems biology. 2013 10;9:693 10.1038/msb.2013.52 24084808 PMC3817402

[20] Kim J, Reed JL. RELATCH: relative optimality in metabolic networks explains robust metabolic and regulatory responses to perturbations. Genome biology. 2012 7;13(9):R78 10.1186/gb-2012-13-9-r78 23013597 PMC3506949

[21] Saha R, Chowdhury A, Maranas CD. Recent advances in the reconstruction of metabolic models and integration of omics data. Current opinion in biotechnology. 2014 10;29:39–45. 10.1016/j.copbio.2014.02.011 24632194

[22] Kim J, Reed JL. Refining metabolic models and accounting for regulatory effects. Current opinion in biotechnology. 2014 10;29:34–38. 10.1016/j.copbio.2014.02.009 24632483

[23] Facchetti G. *Computational approaches to complex biological networks*. PhD thesis, SISSA, Trieste; 2013.

[24] Facchetti G. Greedy and recursive resilencings drive the long-term dynamics of metabolic adaptation. preprint. 2015;.

[25] Orth JD, Fleming RMT, Palsson BO. Reconstruction and Use of Microbial Metabolic Networks: the Core Escherichia coli Metabolic Model as an Educational Guide. EcoSal. 2009. 10.1128/ecosalplus.10.2.1 26443778

[26] Nowak MA. Evolutionary Dynamics. Harvard University Press; 2006 Available from: http://books.google.se/books?id=YXrIRDuAbE0C.

[27] Cornelius SP, Lee JS, Motter AE. Dispensability of Escherichia coli’s latent pathways. Proceedings of the National Academy of Sciences. 2011;108(8):3124 10.1073/pnas.1009772108 PMC3044393 21300895

[28] Schuetz R, Kuepfer L, Sauer U. Systematic evaluation of objective functions for predicting intracellular fluxes in Escherichia coli. Mol Sys Biol. 2007;3:119. 10.1038/msb4100162 PMC1949037 17625511

[29] Schuetz R, Zamboni N, Zampieri M, Heinemann M, Sauer U. Multidimensional optimality of microbial metabolism. Science. 2012 5;336(6081):601–604. 10.1126/science.1216882 22556256

[30] Holzhütter HG. The principle of flux minimization and its application to estimate stationary fluxes in metabolic networks. European journal of biochemistry / FEBS. 2004 7;271(14):2905–2922. 10.1111/j.1432-1033.2004.04213.x 15233787

[31] Nishikawa T, Gulbahce N, Motter AE. Spontaneous reaction silencing in metabolic optimization. PLoS computational biology. 2008 12;4(12):e1000236 10.1371/journal.pcbi.1000236 19057639 PMC2582435

[32] Beard DA, Babson E, Curtis E, Qian H. Thermodynamic constraints for biochemical networks. Journal of Theoretical Biology. 2004;228(3):327–333. 10.1016/j.jtbi.2004.01.008 15135031

