# Supplementary material for: Metabolic Adaptation Processes That Converge to Optimal Biomass Flux Distributions
Source: PLoS Comput Biol. 2015 Sep 4;11(9):e1004434. doi: 10.1371/journal.pcbi.1004434 (PMC4560427; doi:10.1371/journal.pcbi.1004434)
Supplement: S1 text — Details of the population dynamics model and of the selection probabilities. Further considerations of the method and on its applicability. (PDF) [file pcbi.1004434.s001.pdf]

# SUPPLEMENTARY INFORMATION
for
Metabolic adaptation processes that converge to optimal biomass flux distributions

Claudio Altafini
Division of Automatic Control, Dept. of Electrical Engineering,
Linköping University, SE-58183, Sweden

Giuseppe Faccehtti
John Innes Centre, Norwich, NR4 7UH, UK

August 14, 2015

## Contents

**1 Materials** **3**

**2 Methods** **3**
2.1 Population dynamics model for the selection probabilities of the enzyme resilencing. . . . . . . . . 3
2.2 Choosing the "temperature" . . . . . . . . 8

**3 Further considerations on the method and on its applicability.** **9**

# List of Figures

A Metabolic adaptation: convergence of the vector of fluxes to the pFBA optimum . . . . . 13
B Selection probabilities resulting from the replicator equation . . . . . 14
C Time evolution of the correlation-growth 3D histogram . . . . . 15
D Sample trajectories (1) . . . . . 16
E Sample trajectories (2) . . . . . 17
F Sample trajectories (3) . . . . . 18
G Length of a trajectory and number of active reactions vs. growth rate 19
H Average correlation during adaptation . . . . . 20
I Average growth during adaptation . . . . . 21
J Correlation vs. growth rate, with 1/3 of irreversible reactions made reversible . . . . . 22
K Correlation vs. growth rate, with 1/2 of irreversible reactions made reversible . . . . . 23
L Correlation vs. growth rate, with all reversible reactions . . . . . 24
M "Nominal" versus random initial conditions: correlation between the end-points of the trajectories . . . . . 25
N Average number of active bounds during adaptation . . . . . 26
O Average number of active lower and upper bounds per pathway during adaptation . . . . . 27
P Average number of active reactions per pathway during adaptation . 28
Q Correlation vs. growth rate, with original lower and upper bounds . . 29
R Norm of **v** vs. growth rate, with original lower and upper bounds . . 30
S Average number of active bounds during adaptation, with original lower and upper bounds . . . . . 31
T Correlation vs. growth rate. Best trajectory . . . . . 32

# 1 Materials

The metabolic network used in this paper reproduces the central carbon metabolism of the *Escherichia.coli* bacterium. It is described in [1] and downloadable from `http://systemsbiology.ucsd.edu/Downloads/EcoliCore`. It comprises 95 metabolic reactions among 72 metabolites. 49 of these reactions are considered irreversible. The pathways included in this network are Glycolysis/Gluconeogenesis, Pentose Phosphate pathway, TCA cycle, Oxidative Phosphorylation and Pyruvate metabolism, plus a number of exchange reactions.

We assume to be working with wild type strains of *E.coli* growing exponentially in a rich medium, in aerobic conditions. In the network downloaded from `http://systemsbiology.ucsd.edu/Downloads/EcoliCore`, the lower and upper bounds of all reactions are arbitrarily fixed to be equal to 0 or to $\pm 1000$. Following [2], in this paper the bounds are corrected to fit the experimental substrate uptake rates of all single carbon sources described in [3]. The lower bounds of essential chemicals such as oxigen, water, phosphate and ammonia are left sufficiently big so that these compounds never saturate.

# 2 Methods

In what follows the stochastic process used in the paper to describe metabolic adaptation is described in detail. The idea of using resilencings and adjustments to describe metabolic adaptation was first proposed in [4], see also [2]. Here it is presented in a more rigorous mathematical form using replicator equations. It is shown that if the selection probabilities of the resilencings are computed through basic replicator equations, then they naturally assume the form of a Boltzmann distribution. The possibility of including enzyme regulation in the model is explored. This can be done by modulating the shape of the prior probabilities (i.e., the initial conditions of the replicator equation).

## 2.1 Population dynamics model for the selection probabilities of the enzyme resilencing.

In this subsection we describe the population dynamics model used for the estimation of the resilencing probabilities, i.e. the transition probabilities of the resulting

Markov chain.

As explained in the Methods section of the paper, at step $k$ we consider the flux vector $\mathbf{v}_{k-1}$ and the $n_k + 1$ possible phenotypes that can be obtained from it by silencing a single reaction ($n_k$ possibilities, denoted $\mathbf{v}_{k,i}$, $i = 1, \dots, n_k$) or by maintaining unaltered the current flux vector ($\mathbf{v}_{k,0} = \mathbf{v}_{k-1}$). Denote $q_{k,i}$, $i = 1, \dots n_k$, the populations of the $n_k$ possible phenotypes $\mathbf{v}_{k,i}$, and $q_{k,0}$ the population of the current phenotype $\mathbf{v}_{k,0}$. In the interval $\tau \in [0, \Delta t]$, each phenotype has a growth rate $g_{k,i}$ which can be computed from the flux distribution $\mathbf{v}_{k,i}$ using the growth rate equation $g_{k,i} = \xi^T \mathbf{v}_{k,i}$. Viable phenotypes have $g_{k,i} > 0$ while non-viable phenotypes (e.g. when an essential reaction is suppressed) have $g_{k,i} = 0$. These growth rates can be placed on the diagonal of a fitness matrix

$$G_k = \begin{bmatrix} g_{k,0} & & & \\ & g_{k,1} & & \\ & & \ddots & \\ & & & g_{k,n_k} \end{bmatrix}$$

where $g_{k,0}$ represents the growth rate of the current phenotype $\mathbf{v}_{k,0}$.

**Population dynamics modulate selection probabilities. Case 1: uniform priors.** The simplest possible case of a dynamics is given by a replicator equation with initial conditions that are uniformly distributed. Consider the following ODE for the population dynamics in the interval $[0, \Delta t]$:

$$\dot{\mathbf{q}}_k = G_k \mathbf{q}_k \qquad \tau \in [0, \Delta t], \tag{S1}$$

where

$$\mathbf{q}_k = \begin{bmatrix} q_{k,0} \\ q_{k,1} \\ \vdots \\ q_{k,n_k} \end{bmatrix}.$$

Solving (S1):

$$\mathbf{q}_k(\tau) = e^{G_k \tau} \mathbf{q}_k(0) \qquad \tau \in [0, \Delta t]. \tag{S2}$$

The ODE (S1) for the growth of populations in the interval $[0, \Delta t]$ can be normalized to represent probabilities (or frequencies) by using the a replicator equation [5]. The

frequencies (or probabilities) $p_{k,i}$, $i = 0, 1, \ldots, n_k$, associated to the populations $q_{k,i}$, $i = 0, 1, \ldots, n_k$, obey the replicator equation

$$\dot{\mathbf{p}}_k = G_k \mathbf{p}_k - \phi(\mathbf{p}_k)\mathbf{p}_k \qquad \tau \in [0, \Delta t], \tag{S3}$$

where

$$\mathbf{p}_k = \begin{bmatrix} p_{k,0} \\ p_{k,1} \\ \vdots \\ p_{k,n_k} \end{bmatrix}.$$

By $\phi_k$ we denote the so called *average fitness* defined as

$$\phi_k(\mathbf{p}_k) = \sum_{i=0}^{n_k} g_{k,i} p_{k,i}$$

where

$$p_{k,i} = q_{k,i} e^{-\psi_k},$$

with

$$\psi_k(\tau) = \int_0^\tau \phi_k(\mathbf{p}_k(s))ds \qquad \tau \in [0, \Delta t].$$

By construction, $p_{k,i} \geq 0$ and $\|\mathbf{p}_k(\tau)\|_1 = \sum_{i=0}^{n_k} p_{k,i}(\tau) = 1$, $\forall \tau \in [0, \Delta t]$. In particular, it is well-known that $e^{\psi_k(\tau)}$ has the meaning of total population at time $t$:

$$e^{\psi_k(\tau)} = \sum_{i=0}^{n_k} q_{k,i}(\tau) = q_{k,tot}(\tau) \qquad \tau \in [0, \Delta t], \tag{S4}$$

implying that $p_{k,i}(\tau)$ is indeed a frequency:

$$p_{k,i}(\tau) = \frac{q_{k,i}(\tau)}{q_{k,tot}(\tau)}, \qquad i = 0, 1, \ldots, n_k \tag{S5}$$

as expected. In (S4) we can use the explicit expression (S2) for the populations and have then

$$q_{k,tot}(\tau) = \mathbf{1}^T e^{G_k \tau} \mathbf{q}_k(0) = \sum_{i=0}^{n_k} e^{g_{k,i}\tau} q_{k,i}(0) \tag{S6}$$

where $\mathbf{1} = [1 \dots 1]^T$. A straightforward calculation leads to

$$\mathbf{p}_k(\tau) = \mathbf{q}_k(\tau) e^{-\psi_k(\tau)} = e^{-\psi_k(\tau)} e^{G_k \tau} \mathbf{q}_k(0) \qquad \tau \in [0,\, \Delta t], \tag{S7}$$

where the last expression follows from $e^{-\psi_k(\tau)}$ being scalar and hence commuting with the matrix exponential $e^{G_k\tau}$. From (S7): $\mathbf{p}_k(0) = \mathbf{q}_k(0)$, hence (S6) becomes

$$q_{k,tot}(\tau) = \sum_{i=0}^{n_k} e^{g_{k,i}\tau} p_{k,i}(0).$$

If we now assume that the prior probabilities $p_{k,i}(0)$ are uniform (i.e., $p_{k,i}(0) = \frac{1}{n_k+1}$) then $q_{k,tot}(\tau) = \frac{Z_k(\tau)}{n_k+1}$, where $Z_k(\tau) = \sum_{i=0}^{n_k} e^{g_{k,i}\tau}$ is formally analogous to a partition function, in which the fitness $g_{k,i}$ plays the role of the Hamiltonian (or energy function). Therefore, from (S5),

$$p_{k,i}(\tau) = \frac{\frac{e^{g_{k,i}\tau}}{n_k+1}}{\frac{Z_k(\tau)}{n_k+1}} = \frac{e^{g_{k,i}\tau}}{Z_k(\tau)}, \qquad \tau \in [0,\, \Delta t], \qquad i = 0,\, 1, \dots, n_k \tag{S8}$$

i.e., the phenotypes grow with a fitness landscape given by their growth rate. In our Markov chains, at the $k$-th step of one of the trajectories, a phenotype can be selected according to the probabilities $\mathbf{p}_k(\Delta t)$ achieved at the end of the time interval.

In an equation like (S8), the width of the interval, $\Delta t$, plays the role of an inverse temperature (indeed we have $\Delta t = \beta = 1/k_B T$). In particular, in the "low temperature regime" (i.e., when $\Delta t$ is high and so is $\beta$) the frequencies $p_{k,i}(\Delta t)$ concentrate on the fittest phenotypes. The choice becomes deterministic when $\Delta t \to \infty$, i.e. $T \to 0$. In the "high temperature regime" (i.e., when $\Delta t$ is low) the $p_{k,i}(\Delta t)$ tend to resemble the initial uniform distribution, i.e., even viable but low fitness phenotypes have nonnegligible frequencies. At the end of each time interval, the "final" frequencies $\mathbf{p}_k(\Delta t)$ describe how the fitness landscape modulates the probability of silencing from the uniform distribution present at the begin of each interval, see Fig. B for a sketch.

**Population dynamics modulate selection probabilities. Case 2: non-uniform priors.** Just like the activation of a large number of latent pathways in

response to a perturbation can be considered a regulatory program, so it is plausible that one (or more) enzyme regulation strategies are at work in the adaptation phase. In our model we have avoided including any type of mechanistic enzyme regulation information (which could be transcriptional: gene expression regulation, gene mutation, gene context, reaction grouping [6], or post-transcriptional: metabolic sensing and feedback [7], allosteric effects, phosphorylation/dephosphorylation) because no systematic knowledge is available and, even when experimental data can be used, it is difficult to turn this information into an accurate proxy for enzyme activity. It is anyway likely that these enzyme regulatory programs have been shaped by evolution so as to favor the recovery of the growth rate, and contributing significantly to this recovery. Even avoiding sophisticated models of the regulatory "causes" of the improved growth rate, it is possible to improve the plausibility of the model by including a description of the "effects" (i.e., the growth rate itself). This can be accomplished by modulating the prior selection probabilities at the begin of the time interval, $\mathbf{p}_k(0)$, for example choosing them not from a uniform distribution but from a Boltzmann distribution. Then the expressions for $\mathbf{p}_k$ or $\mathbf{q}_k$ can be split into two components, one dynamical (due to the time evolution $\Delta t$), the other instead already active at the begin of each time interval (indicated below as $\beta_k$). Since $G_k$ is diagonal, the exponential of a sum factorizes, and the order of the two factors can be commuted freely:

$$e^{G_k(\Delta t+\beta_k)} = e^{G_k\Delta t}e^{G_k\beta_k} = e^{G_k\beta_k}e^{G_k\Delta t},$$

meaning that considering a single exponential of exponent $\Delta t + \beta_k$ is equivalent to splitting the contribution due to $\Delta t$ from that due $\beta_k$, and incorporating the latter into the prior probabilities at the begin of each interval. Then straightforward calculations give for the new priors (i.e., in $\tau = 0$):

$$\mathbf{q}_k(0) = e^{G_k\beta_k}\mathbf{1} \,/\, (n_k + 1)$$

and

$$\mathbf{p}_k(0) = \mathbf{q}_k(0) \,/\, \mathbf{q}_{k,tot}(0) = e^{G_k\beta_k}\mathbf{1} \,/\, Z_k^{\text{nu}}(0)$$

where $Z_k^{\text{nu}}(0)$, the partition function for the new priors ("nu"=non-uniform), is

$$Z_k^{\text{nu}}(0) = \mathbf{1}^T e^{G_k\beta_k}\mathbf{1} = \sum_{i=0}^{n_k} e^{g_{k,i}\beta_k}.$$

Along the time evolution $[0, \Delta t]$, then, the populations and selection probabilities become

$$\mathbf{q}_k(\tau) = e^{G_k \tau} \mathbf{q}_k(0) = e^{G_k(\tau+\beta_k)} \mathbf{1} \,/\, (n_k + 1), \qquad \tau \in [0, \Delta t]$$

and

$$\mathbf{p}_k(\tau) = \mathbf{q}_k(\tau) \,/\, \mathbf{q}_{k,tot}(\tau) = e^{G_k(\tau+\beta_k)} \mathbf{1} \,/\, Z_k^{\mathrm{nu}}(\tau), \qquad \tau \in [0, \Delta t]$$

where

$$Z_k^{\mathrm{nu}}(\tau) = \mathbf{1}^T e^{G_k \beta_k} e^{G_k \tau} \mathbf{1} = \sum_{i=0}^{n_k} e^{g_{k,i}(\tau+\beta_k)}, \qquad \tau \in [0, \Delta t].$$

Since $Z_k^{\mathrm{nu}}(\tau) = Z_k(\beta_k + \tau)$, the selection probabilities at time $\tau$ still have the form of a Boltzmann distribution

$$p_{k,i}(\tau) = \frac{e^{g_{k,i}(\beta_k+\tau)}}{Z_k(\beta_k + \tau)}, \qquad \tau \in [0, \Delta t] \tag{S9}$$

as it is straightforward to check.

## 2.2 Choosing the "temperature"

As mentioned above, two factors can be used to modulate the amount of randomness in the selection probabilities $\mathbf{p}_k(\Delta t)$ of the resilencings: the length of the time interval $\Delta t$ and the contribution of the prior probabilities $\mathbf{p}_k(0)$ (i.e., the parameter $\beta_k$). Denoting $\beta_k^{\mathrm{glo}} = \beta_k + \Delta t$, then $\beta_k^{\mathrm{glo}}$ has still the interpretation of an inverse temperature (in a Boltzmann equation: $\beta_k^{\mathrm{glo}} = \frac{1}{\kappa_B T}$, where $\kappa_B$ = Boltzmann constant and $T$ = temperature at the $k$-th step). In particular, when $\beta_k^{\mathrm{glo}} \to \infty$, at step $k$ the probability tends to concentrate on the highest growing phenotype, while when $\beta_k^{\mathrm{glo}} \to 0$ all phenotypes become equiprobable.

If we interpret the prior frequency $\mathbf{p}_k(0)$ as the contribution of the regulatory machinery to the activity of the enzymes, then it is plausible to assume that at the early steps of the adaptation (i.e., right after a perturbation has acted on the system disrupting its flux distribution), its importance is dominant over the time evolution of the replicator equation, i.e., $\beta_k \gg \Delta t$ for $k$ small. However it is equally plausible that the driving of enzyme regulation declines with time, and that the effect of the dynamics in differentiating the phenotypes becomes more relevant at later times i.e., $\beta_k < \Delta t$ for $k$ sufficiently large. If for instance we assume that $\Delta t$ is kept constant

throughout the evolution, then we can summarize these considerations by saying that $\beta^{\rm glo}_{k_1} > \beta^{\rm glo}_{k_2}$ for $k_1 < k_2$, see Fig. B.

In the computation of our trajectories for the *E.coli* central metabolism, we have observed that the "cooling" (or, more properly, "heating") schedule adopted is not crucial for reaching the dominant attractor with a high probability, at least as long as $\beta^{\rm glo}_k$ remains sufficiently high. In practice, also focusing on the deterministic case ($\beta^{\rm glo}_k = \infty$, i.e., always selecting the best phenotype) is a good strategy. For what could be explored numerically, initial conditions highly sensitive to early choices (such as the one reported in the bottom row of Fig. F) appear to be quite rare, at least among the $\mathbf{v}$ achieving a high growth.

# 3 Further considerations on the method and on its applicability.

**Core vs. full metabolic network: a question of computational complexity (but not only).** All results of this paper have been obtained using the core metabolic network of *E.coli* which, despite its relatively small number of reactions, contains already many of the features typical of the full *E.coli* metabolic network (robustness, redundancy, loops, characteristic motifs, etc.). It is worth observing that restriction to the central carbon metabolism is a common assumption in many studies dealing with computationally intense methods (e.g. [8, 9]). Scaling our method to the full *E.coli* metabolic network (which has about 2000 reactions [10]) requires extensive computational means (calculating a single Markov chains requires $\approx$ 2 days on a 2.3 GHz CPU). A limited number of trials seems to indicate that growth can be recovered only to some extent, and so does alignment with $\mathbf{v}^{\rm pFBA}$. If instead of $\mathbf{v}^{\rm pFBA}$ computed on the original polytope $\Gamma$, one looks at the flux vector $\mathbf{v}^{\rm pFBA}$ computed on the current $\Gamma_k$ (with $k$ reactions silenced) then the results are much more impressive and close enough to those of the core metabolic network.

It remains to be investigated if extending the idea of sequential resilencings from the central carbon metabolism to the entire metabolic network is an acceptable hypothesis, or if it is more reasonable to focus the resilencings on certain classes of pathways, like those related to energy and to redox metabolism. The metabolic profiling of [11] and [12] for example indicates that environmental perturbations affect primarily the early response of the central metabolism, and that it is a con-

sistent decrease of fluxes on energy-demanding pathways such as glycolysis, pentose phosphate pathway and TCA cycle that lead to temporary growth arrest. For trajectories computed on the entire network, it is likely that the irreversibility of a silencing becomes a restrictive assumption. For example, the transcriptional machinery is almost halted right after the perturbation, but needs to be restarted as the growth rate recovers, which require certain metabolic pathways (like nucleotide and aminoacid biosynthesis) to be fully functional regardless of previous silencings. It is for example shown in [11] that the growth arrest that follows various stress responses does not forbid these metabolites to accumulate, meaning that the corresponding reactions are not halted. Little is known experimentally for most other pathways.

**Reactions that saturate during adaptation.** In our trajectories, a number of bounds become saturated during adaptation. If we group the trajectories based on the growth achieved at the end-point, then a significant difference can be observed between low-growth and high-growth trajectories, see Fig. N. In particular, the latter tend to saturate a larger number of bounds in the early phase of the adaptation. It is possible to separate lower from upper bounds and to break down further these two classes into the specific pathways that compose the central metabolism, see Fig. O. When looking at the lower bounds, one category neatly emerges, that of exchange reactions. For high-growth phenotypes, the uptake bounds become active in the early steps and stay active until around step 10. If we look at specific reactions, then it is possible to see that uptake of glucose, fructose are nearly always saturated, and so are succinate, fumarate and malate (entering into the TCA cycle). Also substrates related to nitrogen metabolism, such as glutamine and glutamate, are highly saturated. As for the upper bounds, two pathways saturate with high significance: pyruvate metabolism and transport reactions, notably the secretion of ethanol, pyruvate and 2-oxoglutarate. Taken together, these active constraints suggest that in order to achieve maximal growth the cell has to be able to efficiently use resources at the early stages of the adaptation. When instead in the first steps a trajectory remains "internal" to the polytope $\Gamma_k$ then it will settle to a lower growth once adapted.

**Influence of bound values of the metabolic network on adaptation** As mentioned in the Material section, with respect to the standard *E.coli* core network

downloadable from `http://systemsbiology.ucsd.edu/Downloads/EcoliCore`, in our model the lower and upper bounds of the reactions have been rendered more realistic using the experimental supply uptake rate provided in [3]. This choice has a minor influence on the quality of the results. As a matter of fact, using the original bounds provided with the model (all, except ATP maintenance, are 0 or ±1000), while the growth rate achieved by the adaptation processes worsens considerable, the correlation at absorption, $\mathrm{corr}(\mathbf{v}^{\mathrm{pFBA}}, \mathbf{v})$, is only slightly worse. Details are given in Fig. Q and Fig. R. Statistics for this case are computed over $2 \cdot 10^4$ trajectories. The difference is due to the original bounds being more "artificial" than those adopted here, and leading more frequently to trajectories that remain internal in the polytope $\Gamma_k$, see Fig. S. In spite of the reduced growth what is important is that the alignment of $\mathbf{v}$ on $\mathbf{v}^{\mathrm{pFBA}}$ is confirmed. Therefore we can conclude that also in this case the normalized pFBA vector, $\frac{\mathbf{v}^{\mathrm{pFBA}}}{\|\mathbf{v}^{\mathrm{pFBA}}\|}$, plays the role of dominant attractor for the metabolic adaptation process.

**Effect of stochasticity on the trajectories.** As shown in Figs. D-F, when repeated trajectories are computed starting from an identical initial condition, then in some cases the stochasticity encoded in the metabolic adaptation process may lead to end-points having different growth and correlation properties. If we think of these trajectories as strains evolving in parallel, then it is plausible that the fittest strain tends to prevail over the others in terms of absolute abundance. If in our Markov chains we keep only the best trajectory for each initial condition, then all statistical parameters improve of a small fraction, see Fig. T. In particular the lack of substantial improvement in the growth rate tells us that most initial conditions have limited sensitivity to the randomness of the Markov chains. This confirms that cases like the bottom row of Fig. F are indeed rare. The low sensitivity to early choices is one of the reasons of the massive convergence we see in our Markov chains. It is a sign of the "structural" robustness of the attractor.

# References


[1] Orth, J, Fleming, R, & Palsson, B. (2009) Reconstruction and use of microbial metabolic networks: the core Escherichia coli metabolic model as an educational guide. *EcoSal* **doi: 10.1128/ecosal.10.2.1**.

[2] Facchetti, G. (2015) Greedy and recursive resilencings drive the long-term dynamics of metabolic adaptation. *preprint.*

[3] Fong, S. S & Palsson, B. Ø. (2004) Metabolic gene–deletion strains of Escherichia coli evolve to computationally predicted growth phenotypes. *Nature genetics* **36**, 1056–1058.

[4] Facchetti, G. (2013) *Computational approaches to complex biological networks.* (PhD thesis, SISSA, Trieste).

[5] Nowak, M. (2006) *Evolutionary Dynamics.* (Harvard University Press).

[6] Park, J, Kim, T, & Lee, S. (2010) Prediction of metabolic fluxes by incorporating genomic context and flux-converging pattern analysis. *Proc. Natl. Acad. Sc.* **107**, 14931–14936.

[7] Kotte, O, Zaugg, J. B, & Heinemann, M. (2010) Bacterial adaptation through distributed sensing of metabolic fluxes. *Molecular systems biology* **6**, 355.

[8] Schuetz, R, Kuepfer, L, & Sauer, U. (2007) Systematic evaluation of objective functions for predicting intracellular fluxes in Escherichia coli. *Mol. Sys. Biol.* **3**, 119.

[9] Schuetz, R, Zamboni, N, Zampieri, M, Heinemann, M, & Sauer, U. (2012) Multidimensional optimality of microbial metabolism. *Science* **336**, 601–604.

[10] Orth, J. D, Conrad, T. M, Na, J, Lerman, J. A, Nam, H, Feist, A. M, & Palsson, B. Ø. (2011) A comprehensive genome-scale reconstruction of Escherichia coli metabolism. *Molecular Systems Biology* **7**, 535.

[11] Jozefczuk, S, Klie, S, Catchpole, G, Szymanski, J, Cuadros-Inostroza, A, Steinhauser, D, Selbig, J, & Willmitzer, L. (2010) Metabolomic and transcriptomic stress response of Escherichia coli. *Molecular Systems Biology* **6**, 364.

[12] Fong, S, Nanchen, A, Palsson, B, & Sauer, U. (2006) Latent pathway activation and increased pathway capacity enable Escherichia coli adaptation to loss of key metabolic enzymes. *Journal of Biological Chemistry* **281**, 8024–8033.

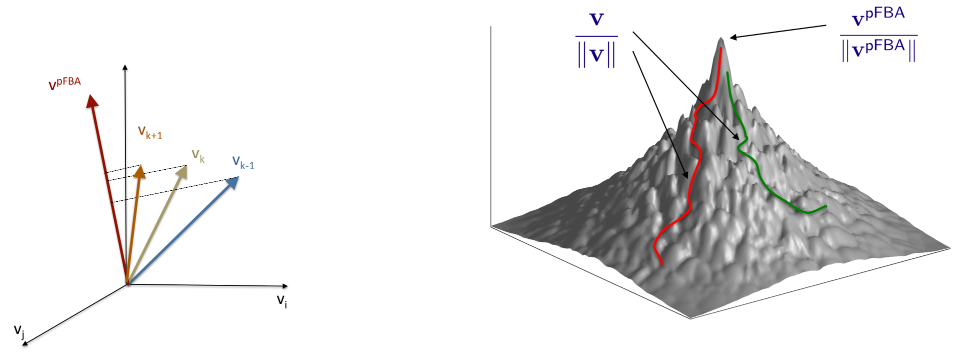


(i) (ii)

Figure A: Metabolic adaptation: convergence of the vector of fluxes to the pFBA optimum. (i): Iterating the resilencing procedure, the correlation between $\mathbf{v}^{\mathrm{pFBA}}$ and $\mathbf{v}_k$ normally increases, i.e., the two vectors tend to become aligned, although they need not have the same length. (ii): For most initial conditions in $\Gamma$, $\frac{\mathbf{v}_k}{\|\mathbf{v}_k\|} \to \frac{\mathbf{v}^{\mathrm{pFBA}}}{\|\mathbf{v}^{\mathrm{pFBA}}\|}$. Many local maxima exist, but they have a very limited basin of attraction.

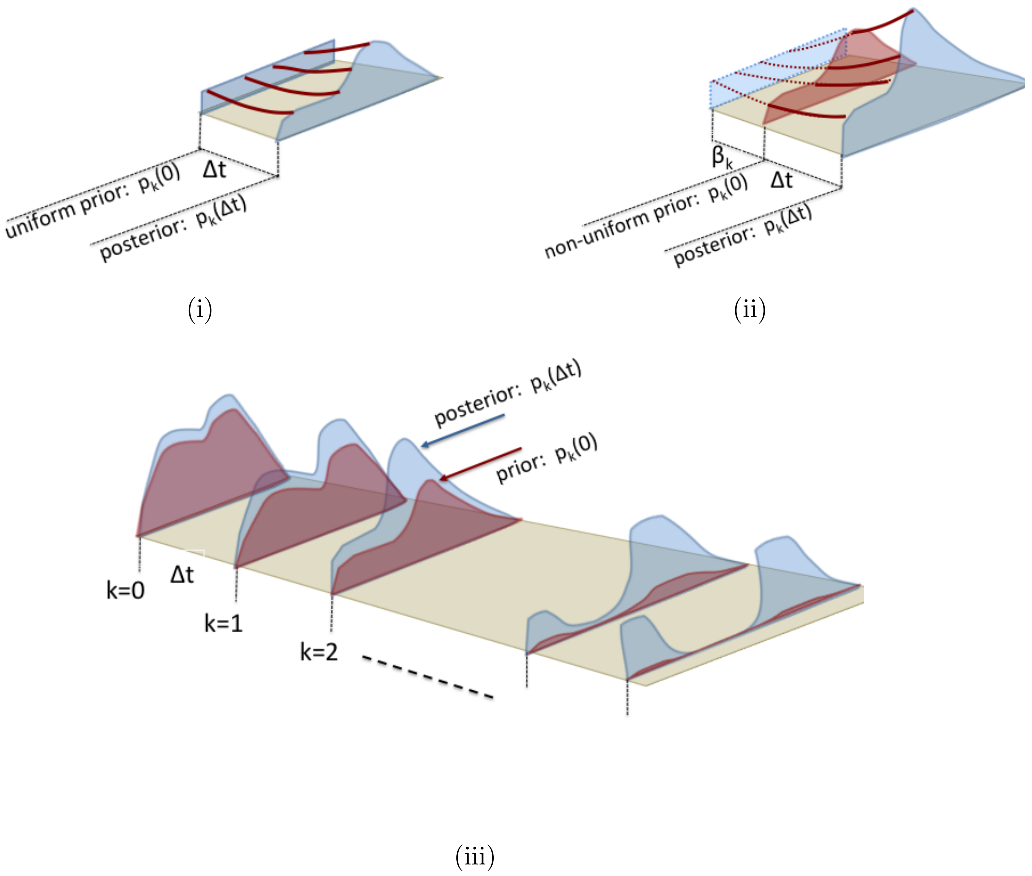


Figure B: Sketch of the selection probabilities resulting from the replicator equation. In (i) uniform priors are used for the probabilities (i.e., the influence of enzyme regulation is considered irrelevant). In (ii) instead the priors are not uniform, but computed through a Boltzmann distribution (representing the effect of the enzyme regulation), effects which adds up with the dynamical evolution. (iii): sketch of a potential procedure for the "temperature" of the global Boltzmann distribution of the selection probabilities. In the early phase of the adaptation the probabilities are more focused on the fittest phenotypes as an enzyme regulatory program strongly influences the selection of the resilencings. At later stages of the adaptation, the influence of the regulatory machinery weakens and selection can become biased towards random events such as gene mutations.

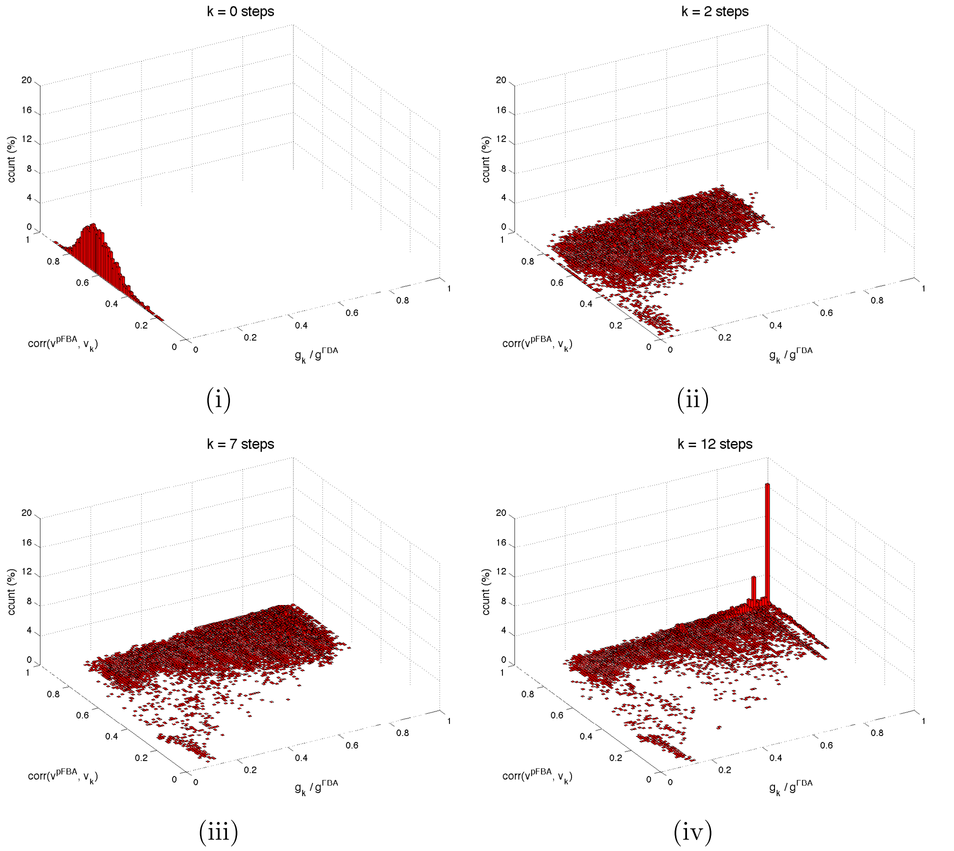


Figure C: Time evolution of the histogram of Fig. 3 during adaptation. The steps $k = 0, 2, 7, 12$ are shown. Starting from 0-growth, and a correlation normally distributed around $\mathrm{corr}(\mathbf{v}^{\mathrm{pFBA}}, \mathbf{v}) \approx 0.6$, the histogram gradually moves towards the high growth / high correlation corner, where most trajectories eventually converge. In doing so, no transient peak emerge, meaning that the trajectories explore a broad number of different resilencing strategies. This is one of the main clues that the domain of attraction of the high growth / high correlation corner is indeed very large.

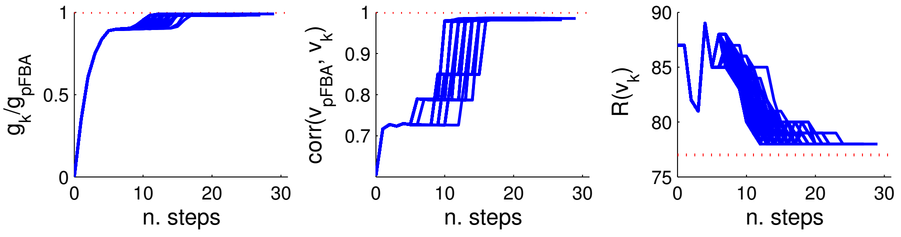


(i)

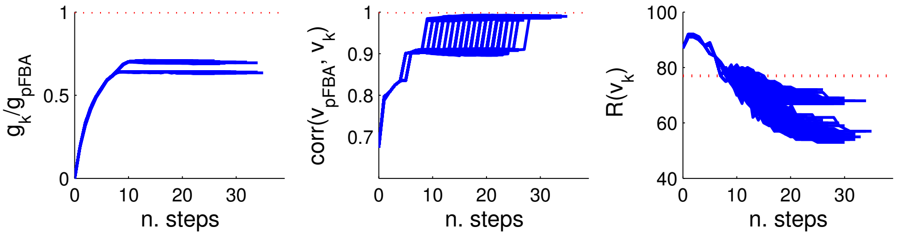


(ii)

Figure D: Time course of 100 trajectories from 2 different initial conditions. The 3 columns of panels show: $g_k/g^{\mathrm{FBA}}$ (left), $\mathrm{corr}(\mathbf{v}^{\mathrm{pFBA}}, \mathbf{v}_k)$ (middle), and number of active reactions $R(\mathbf{v}_k)$ (right). All trajectories of the first row converge to the maximal growth (more precisely to $g/g^{\mathrm{FBA}} > 0.99$), to maximal correlation ($\mathrm{corr}(\mathbf{v}^{\mathrm{pFBA}}, \mathbf{v}) > 0.99$) and to a number of active reactions which is one more than the pFBA (red dotted line) which however does not alter significantly $g$. The path that leads to maximal growth is not unique, in particular in the order of the resilencings. Concerning the second initial condition, no trajectory achieves a growth ratio $g/g^{\mathrm{FBA}}$ bigger than 0.72, although all trajectories achieve a correlation of at least 0.9 (and nearly all of at least 0.98). The influence of two different (nearby) local attractors is visible in the trajectories.

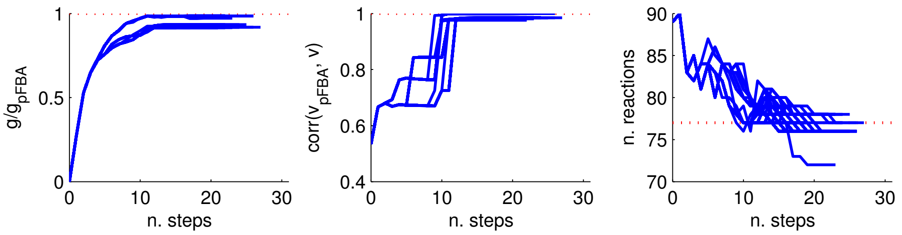


(i)

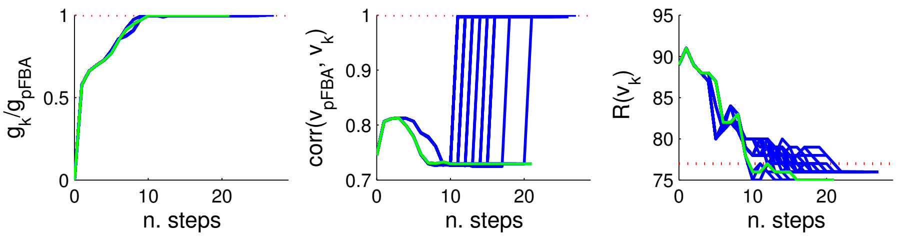


(ii)

Figure E: Two sets of 100 trajectories from 2 different initial conditions. Notation is the same as in Fig. D. In the first row the trajectories reach $\mathbf{v}^{\mathrm{pFBA}}$ or a nearby point of growth rate slightly lower. In the second row, all trajectories achieve maximal growth (up to a negligible difference). However, some of them do not converge to $\mathbf{v}^{\mathrm{pFBA}}$ but to another FBA flux distribution (the green trajectory has a correlation of 0.7 with $\mathbf{v}^{\mathrm{pFBA}}$).

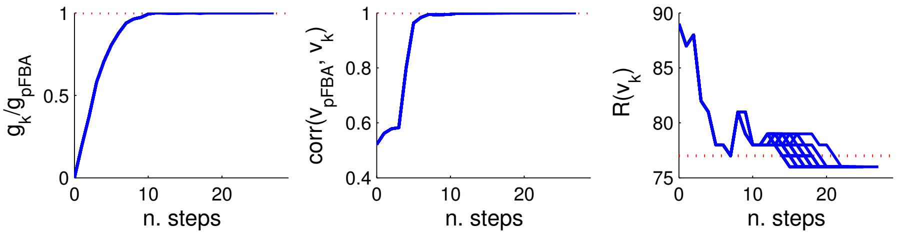


(i)

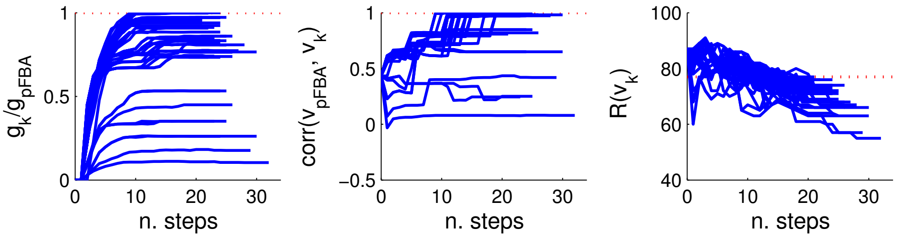


(ii)

Figure F: A few more trajectories reaching the optimum from 2 different initial conditions. In the top row all 100 trajectories are overlapping, i.e., the stochasticity of the selection probability is not enough for the Markov chain to explore more than a single resilencing strategy. In the bottom row, instead, the initial condition is susceptible to follow different routes, and these alternatives are highly sensitive to the early choices. This leads to a wide variety of trajectories, some of which can reach $\mathbf{v}^{\mathrm{pFBA}}$, while the majority does not, leading in some cases to minimal growth and low correlation. While the situation in the top row is quite common, the one in the bottom row is quite rare.

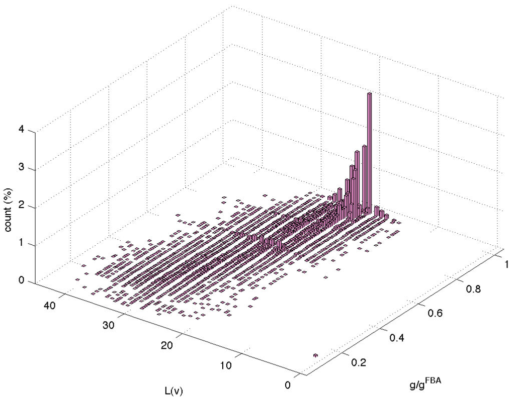


(i)

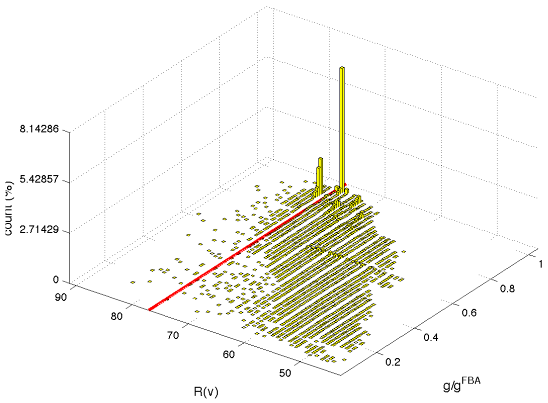


(ii)

Figure G: Length of a trajectory and number of active reactions vs. growth rate. (i): The histogram shows the length $L$ of a trajectory (i.e., the number of steps before absorption into an ergodic state) v.s. the growth rate reached at the end-point for the trajectories of Fig. 3. The trajectories achieving high growth utilize less steps than those attaining a lower growth. (ii): The histograms shows the number of active reactions $R$ in the ergodic states reached by the trajectories. The number of active reactions is essentially proportional to the growth rate. The red line represents the number of active reactions of $\mathbf{v}^{\mathrm{pFBA}}$.

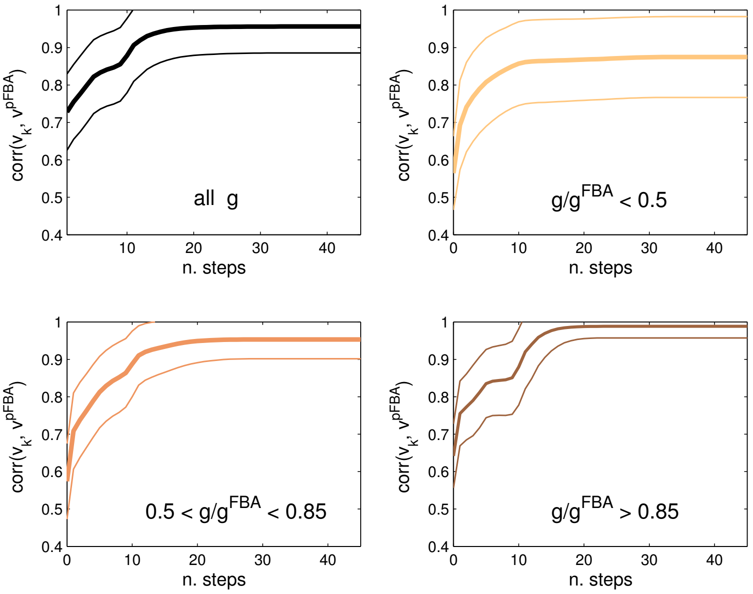


Figure H: Average correlation corr($\mathbf{v}^{\mathrm{pFBA}}, \mathbf{v}_k$) during adaptation, for all trajectories (top-left panel) and for 3 classes of $g$ (achieved at the end-point). Mean $\pm$ standard deviation are shown. The correlation is growing monotonically in all plots.

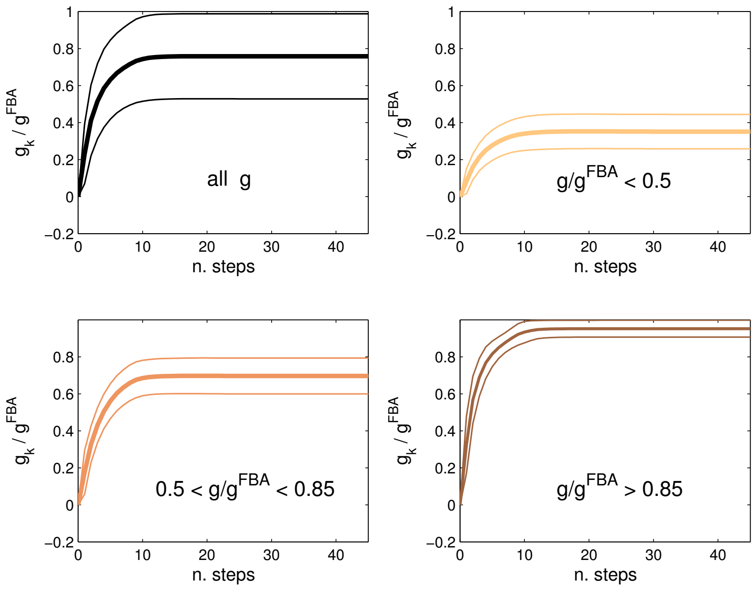


Figure I: Average growth rate during adaptation for all trajectories (top-left panel) and for 3 classes of $g$ (achieved at the end-point). In all plots $g$ is monotonically growing.

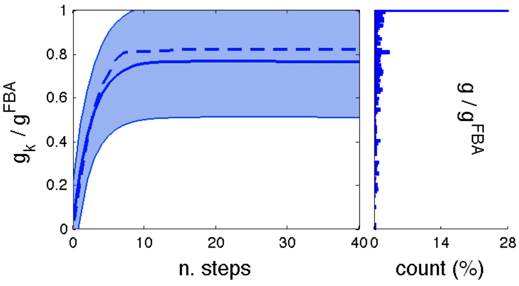


(i)

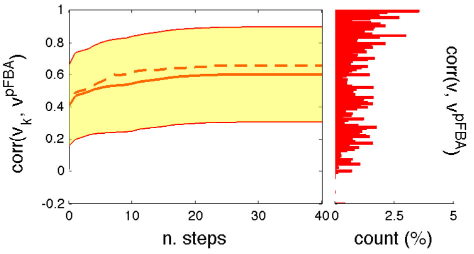


(ii)

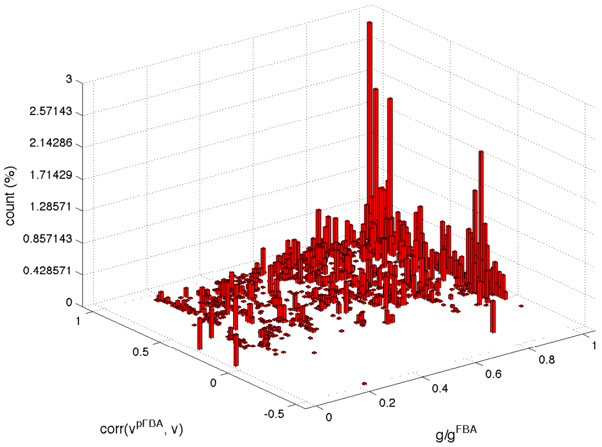


(iii)

Figure J: Correlation vs. growth rate for the *E.coli* metabolic network with 1/3 of irreversible reactions made reversible. When a (randomly chosen) fraction of the irreversible reactions of the metabolic network are made reversible by modifying the corresponding $\ell_i$, then the time profiles for growth $g/g^{\mathrm{pFBA}}$, are still similar to those Fig. 3, but the correlation with $\mathbf{v}^{\mathrm{pFBA}}$ worsens considerably. As can be seen in the 3D histogram, this implies that trajectories reaching maximal growth do not converge all to $\mathbf{v}^{\mathrm{pFBA}}$, but partly also to other degenerate optimal flux distribution $\mathbf{v}^{\mathrm{FBA}}$.

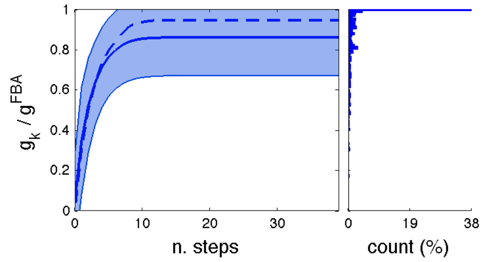


(i)

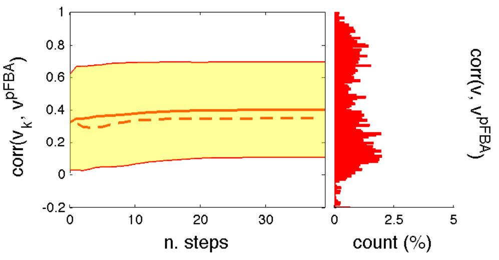


(ii)

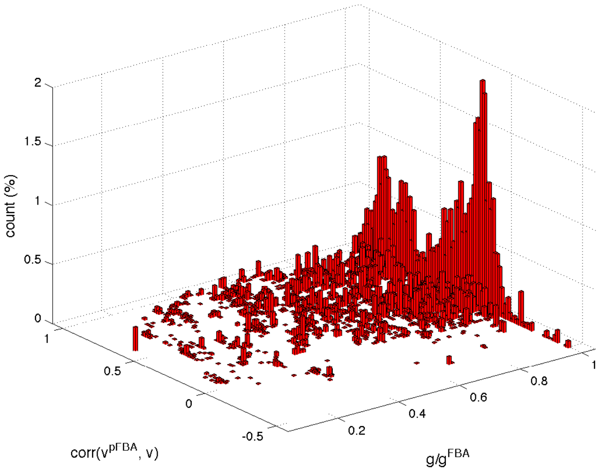


(iii)

Figure K: Correlation vs. growth rate for the *E.coli* metabolic network with 1/2 of irreversible reactions made reversible. When the fraction of the irreversible reactions of the metabolic network which are made reversible by modifying the corresponding $\ell_i$ is increased, then maximal growth is still achieved but the peak of correlation $\mathrm{corr}(\mathbf{v}^{\mathrm{pFBA}}, \mathbf{v})$ disappears, in favor of other flux distributions $\mathbf{v}^{\mathrm{FBA}}$ giving equivalent biomass.

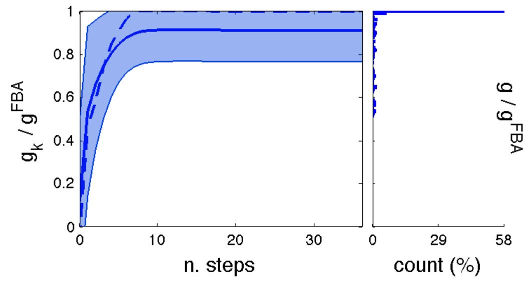


(i)

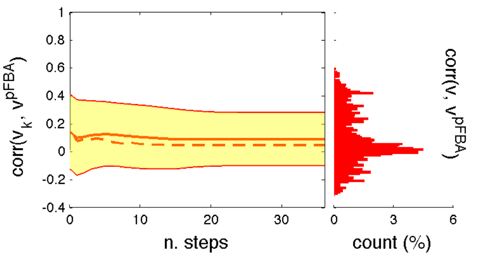


(ii)

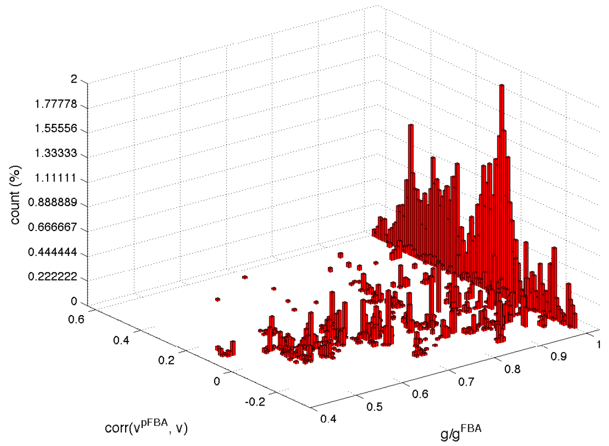


(iii)

Figure L: Correlation vs. growth rate for the *E.coli* metabolic network with all reversible reactions. When all reactions are reversible, the trajectories achieve the best growth rate at absorption with a mean value $\frac{\langle g \rangle}{g^{\mathrm{FBA}}} = 0.91$, a median of 0.997, and 76.4% of trajectories reaching a growth rate of at least $0.85 g^{\mathrm{pFBA}}$. The peak of correlation $\mathrm{corr}(\mathbf{v}^{\mathrm{pFBA}}, \mathbf{v})$ has completely disappeared, and the correlation histogram between $\mathbf{v}$ and $\mathbf{v}^{\mathrm{FBA}}$ is centered at 0. Notice how achieving maximal growth requires now less silencings than with the proper irreversibility constraints of Fig. 3.

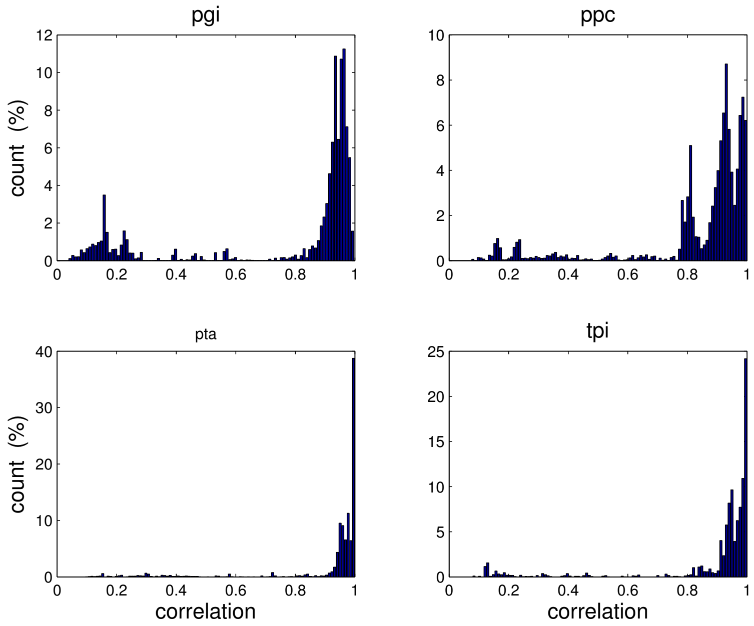


Figure M: "Nominal" versus random initial conditions: correlation between the end-points of the trajectories. Following [12], adaptation of 4 knockout strains of *E.coli* to a switch in substrate from rich medium (Luria-Bertani) to glucose as single carbon source is considered. In this case, following [2], the initial condition of the Markov chains can be computed using the same procedure described in the Methods section of the paper. It can however also be chosen randomly inside $\Gamma$, as in the rest of this paper. When the two types of trajectories (from "nominal" and from random initial conditions) are computed and their terminal point compared, it can be seen that the correlation is always very high (i.e., both types of trajectories tend to converge towards $\mathbf{v}^{\mathrm{pFBA}}$). The *pgi* mutant has a second minor peak at low correlation, corresponding to an alternative phenotype of sub-optimal growth, sometimes observed in both types of trajectories, and documented experimentally in [12].

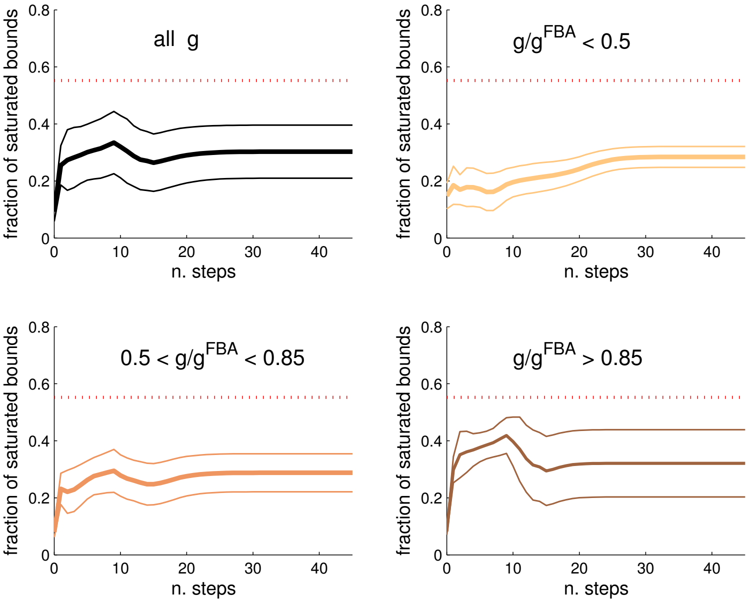


Figure N: Average number of active bounds during adaptation, for all trajectories (top-left panel) and for 3 classes of $g$ (achieved at the end-point). Mean $\pm$ standard deviation are shown. The trajectories achieving low $g$ have a low number of active bounds in the early phase of adaptation (top right panel), while the trajectories that approach $g^{\mathrm{FBA}}$ saturate the most of bounds during the transient.

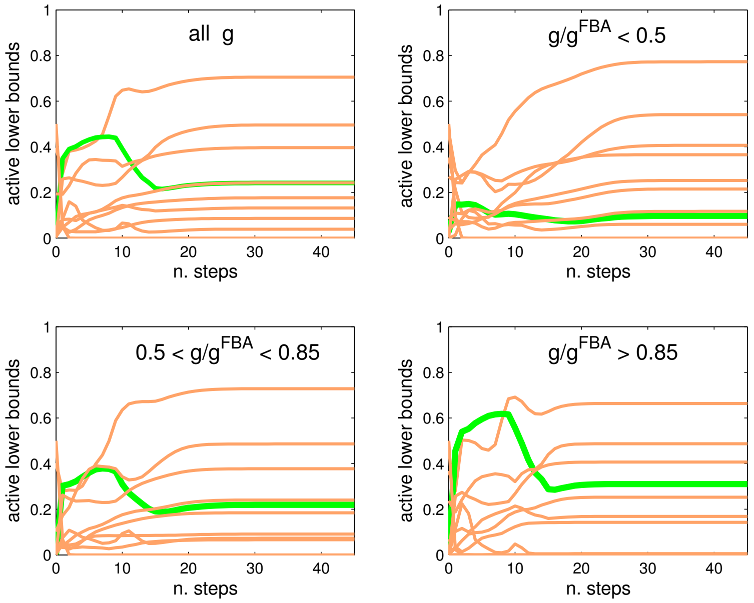


(i)

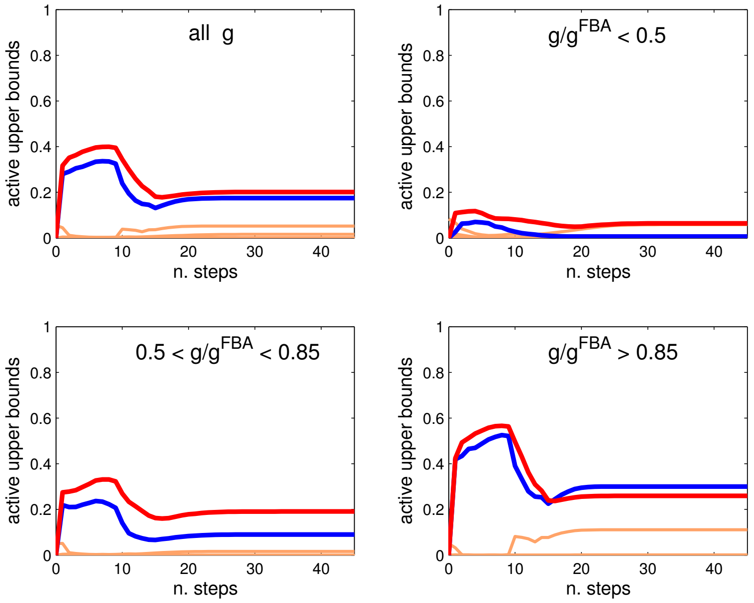


(ii)

Figure O: Average number of active lower (i) and upper (ii) bounds during adaptation, divided by pathway, for all trajectories (top-left panel of each subfigure) and for 3 classes of $g$ (achieved at the end-point). Mean $\pm$ standard deviation are shown. If the active bound of Fig. N are split into lower and upper bounds and subdivided into the various pathways forming the central metabolism, then it can be seen that the substantial difference between high growth and modest growth is due to the saturation of the lower bounds in the exchange reactions (green curve in (i)) and due to the pyruvate metabolism (blue curve in (ii)) and to the transport reactions (red curve in (ii)).

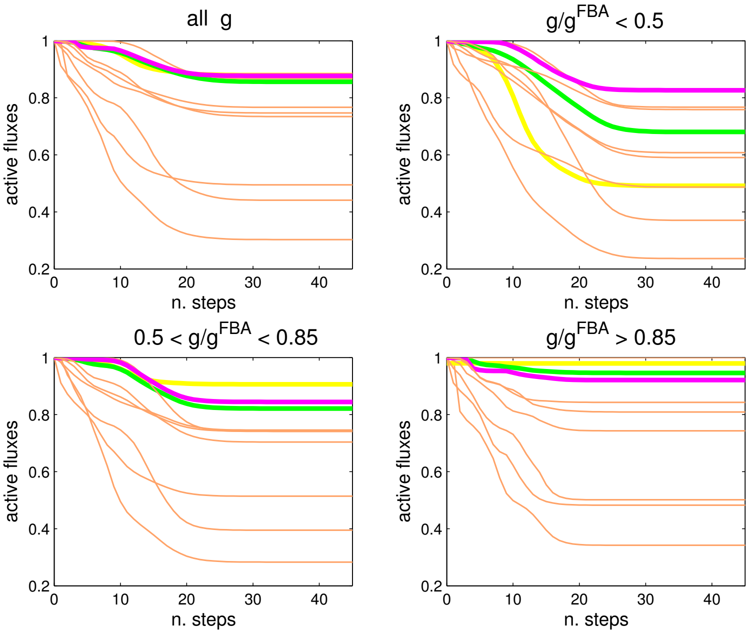


Figure P: Average number of active reactions during adaptation, for all trajectories (top-left panel) and for 3 classes of $g$ (achieved at the end-point). The trajectories achieving high $g$ maintain all (or nearly all) active reactions in the TCA cycle (yellow), pentose phosphate pathway (magenta) as well as all exchange reactions (green). Those leading to poor growth instead tend to underuse these pathways, notably the TCA cycle.

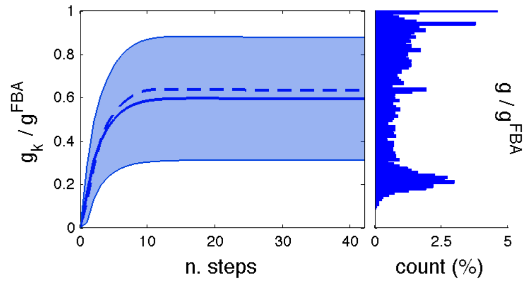


(i)

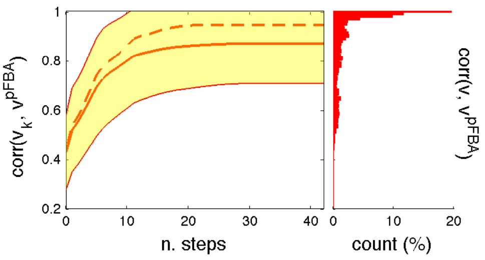


(ii)

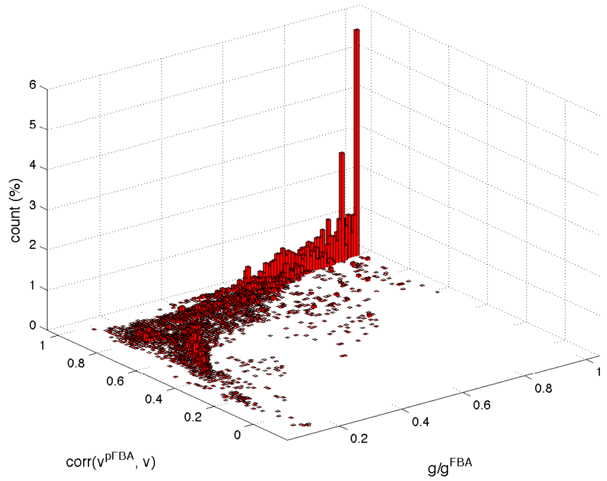


(iii)

Figure Q: Correlation vs. growth rate for the *E.coli* metabolic network with original lower and upper bounds. This network differs from the one used in the current paper in the choice of lower and upper bounds (those used in [1] are more symmetric than ours, and nearly all identical). Comparing with Fig. 3 of the paper, mean and median for the growth rate at absorption are now resp. 0.6 and 0.64, hence they have worsened when compared with Fig. 3. This is mostly due to a large peak at low growth visible in the vertical histogram. For what concerns $\mathrm{corr}(\mathbf{v}^{\mathrm{pFBA}}, \mathbf{v})$, the situation is more similar to what seen in Fig. 3: mean is 0.87 and median is 0.94. The histogram is still significantly skewed towards maximal alignment of $\mathbf{v}$ on $\mathbf{v}^{\mathrm{pFBA}}$, with 68% of end-point above the mean. The pattern of the 3D histogram is still faithful to that of Fig. 3: of the trajectories having $g/g^{\mathrm{pFBA}} > 0.85$, 98.5% have correlation $\geqslant 0.9$.

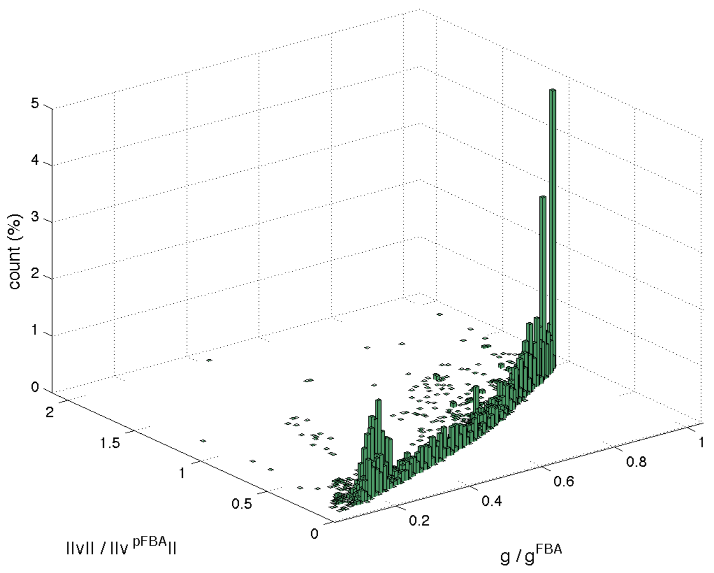


Figure R: Norm of **v** vs. growth rate for the *E.coli* metabolic network with original lower and upper bounds. Analogously to Fig. 4, the growth rate is decided by the norm $||\mathbf{v}||$ at the time of absorption.

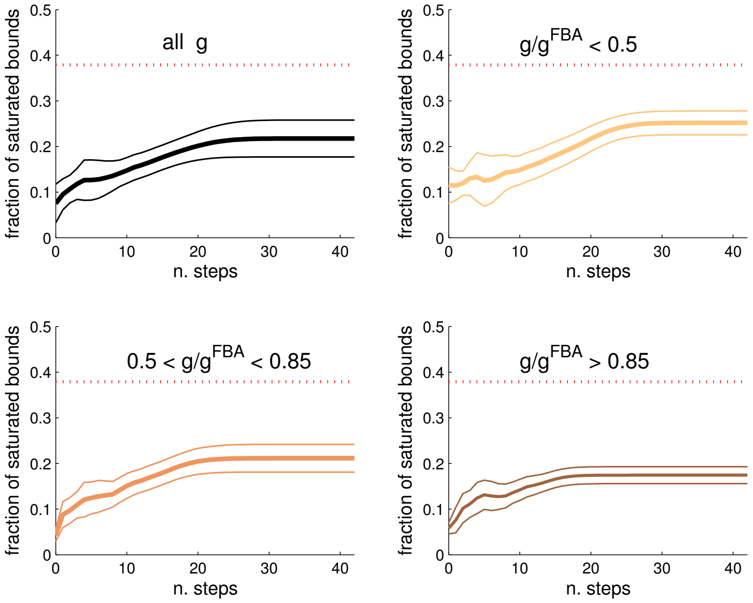


Figure S: Average number of active bounds during adaptation for the *E.coli* metabolic network with original lower and upper bounds, for all trajectories (top-left panel) and for 3 classes of $g$ (achieved at the end-point). Mean $\pm$ standard deviation are shown. Unlike in Fig. N, no substantial difference is visible between the trajectories achieving low $g$ (top right panel) and those that approach $g^{\mathrm{FBA}}$ (bottom right panel).

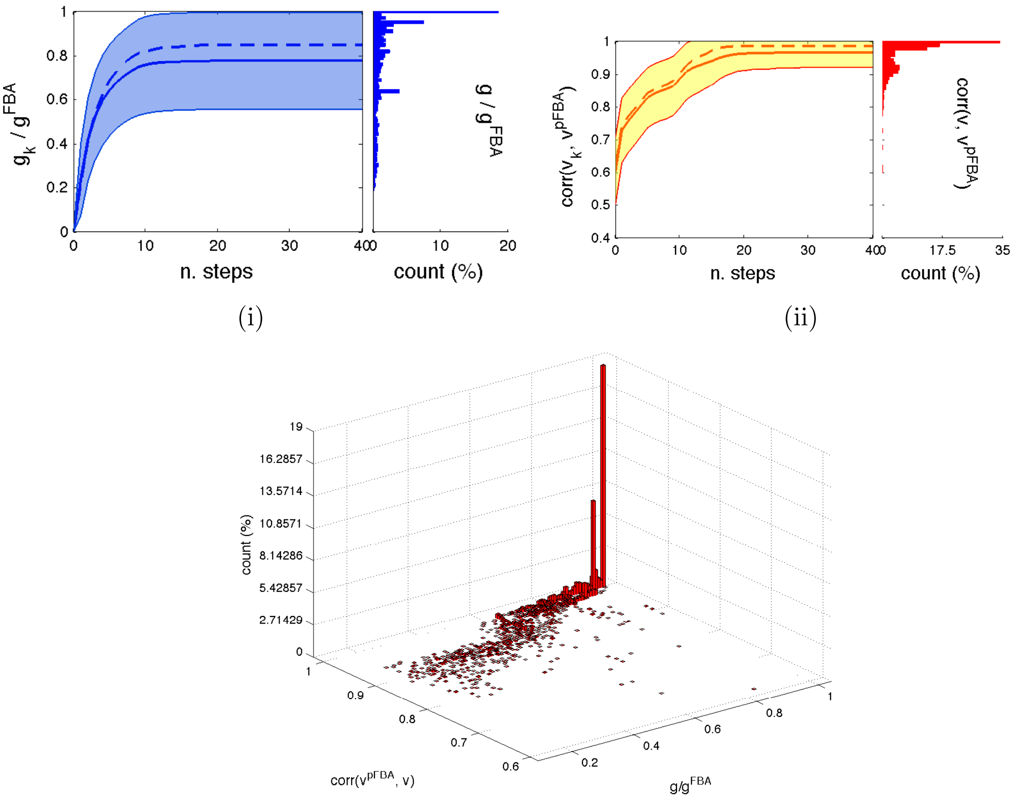


(iii)

Figure T: Correlation vs. growth rate for the *E.coli* metabolic network. Best trajectory for every initial condition. For the resulting $\sim 5000$ trajectories, mean, median and std are slightly improved with respect to those of Fig. 3. 52% reach a growth rate of $0.85g^{\mathrm{pFBA}}$, while 97.6% reach a correlation of at least 0.85 with $\mathbf{v}^{\mathrm{pFBA}}$.
